# Ribosomal protein deficiencies linked to Diamond-Blackfan anemia induce distinctive alterations of ATF4 expression

**DOI:** 10.1016/j.isci.2025.112138

**Published:** 2025-03-01

**Authors:** L. Francisco Lorenzo-Martín, Javier Robles-Valero, Rosa Ramírez-Cota, Sonia G. Gaspar, Pedro Fuentes, Antonio Gentilella, Xosé R. Bustelo, Mercedes Dosil

**Affiliations:** 1 Centro de Investigación del Cáncer, CSIC-University of Salamanca, Campus Unamuno, 37007 Salamanca, Spain; 2 Instituto de Biología Molecular y Celular del Cáncer, CSIC-University of Salamanca, Campus Unamuno, 37007 Salamanca, Spain; 3 Centro de Investigación Biomédica en Red de Cáncer (CIBERONC), CSIC-University of Salamanca, Campus Unamuno, 37007 Salamanca, Spain; 4 Laboratory of Cancer Metabolism, ONCOBELL Program, Bellvitge Biomedical Research Institute (IDIBELL), Barcelona, Spain; 5 Department of Biochemistry and Physiology, Faculty of Pharmacy and Food Science, University of Barcelona, Barcelona, Spain; 6 Departamento de Bioquímica y Biología Molecular, University of Salamanca, Campus Unamuno, 37007 Salamanca, Spain

**Keywords:** Biochemistry, molecular biology, omics

## Abstract

Ribosomal protein haploinsufficiency causes Diamond-Blackfan anemia (DBA) and other ribosomopathies. DBA has been linked to p53 activation and reduced GATA1 expression, but these mechanisms do not fully explain the disease. This study unveils that deficiencies in small (RPS) or large (RPL) ribosomal subunit proteins cause a p53-independent loss of ATF4, a master regulator of stress responses and erythropoiesis, by reducing the pool of actively translating *ATF4* mRNAs. This defect is more pronounced in RPS deficiencies because the loss of 40S, but not 60S, subunits cause a destabilization of *ATF4* transcripts. *ATF4* downregulation occurs in early hematopoietic progenitors and correlates with the severity of erythroid differentiation defects in patients with DBA. It is also linked to the de-repression of fetal hemoglobin in erythroid cells, a frequent feature in patients with DBA. Our findings indicate that impaired *ATF4* expression might be a primary contributor to DBA and explain the aggravated erythroid failure of RPS-mutant patients.

## Highlights

• *ATF4* mRNA levels and translation are highly dependent on ribosome subunit production
• *ATF4* expression is reduced more severely in 40S than in 60S subunit deficiencies
• ATF4 function is compromised in patients with DBA with strong erythropoiesis defects
• Ribosome biogenesis defects induce the de-repression of fetal globin expression

## Graphical abstract

**Figure:**
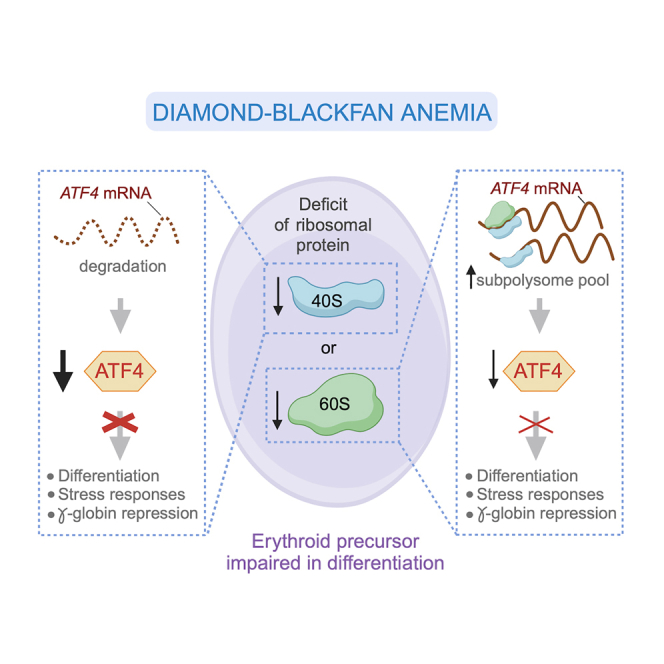

## Introduction

The synthesis of ribosomes, also referred to as ribosome biogenesis, involves the assembly of the 4 ribosomal RNAs (rRNAs) and 80 ribosomal proteins into the small (40S) and large (60S) ribosomal subunits. This process is initiated with the transcription of the initial rRNA precursor (47S pre-rRNA) within the nucleolus that, upon cleavage, renders the precursor for the 18S rRNA (component of the mature 40S ribosomal subunit) and the precursor for both the 28S and 5.8S rRNAs (components of the mature 60S ribosomal subunit). These two pre-rRNA precursors follow independent downstream maturation pathways, each consisting in the stepwise generation of several intermediates (preribosomes) in the nucleolus, nucleoplasm, and cytoplasm.^1^^,^^2^^,^^3^^,^^4^^,^^5^^,^^6^ The proper formation of each preribosome requires the timely incorporation of ribosomal proteins and *trans*-acting ribosome biogenesis factors that mediate specific pre-rRNA processing, rRNA folding, rRNA modification, or ribosomal protein assembly events.^4^ Because each 40S and 60S ribosomal protein is incorporated at a very specific preribosome intermediate, the defective production of one ribosomal protein leads to a specific block in the maturation of its upstream intermediate and the defective production of the downstream ribosomal subunit.^7^ Thus, in the case of a loss of a 40S ribosomal protein, cells show the rapid accumulation of aberrant 40S preribosomes, a blockage in 40S subunit production, and accumulation of free mature 60S subunits. Likewise, the loss of a given 60S ribosomal protein will cause a stop in 60S preribosome maturation, the loss of 60S subunits, and the accumulation of free mature 40S subunits in cells. In both cases, the endpoint result is an impairment in the production of ribosomes.

Although a major loss of ribosomes is lethal for cells, mutations in genes encoding either ribosome biogenesis factors or ribosomal proteins can lead to diseases that are generally known as ribosomopathies.^8^^,^^9^^,^^10^^,^^11^^,^^12^^,^^13^^,^^14^^,^^15^ One of them is DBA, an autosomal dominant heritable disease characterized by erythroid hypoplasia and a heterogeneous spectrum of congenital malformations affecting the craniofacial skeleton, heart, genitourinary system, and limbs.^16^^,^^17^ Patients with DBA are also more prone to cancer.^18^^,^^19^^,^^20^ About 75% of the cases of this disease are caused by heterozygous mutations in genes encoding ribosomal proteins belonging to either the small (i.e., RPS19/eS19, RPS26/eS26) or the large (i.e., RPL5/uL18, RPL11/uL5) ribosomal subunits.^21^^,^^22^

One of the best-known processes involved in the development of DBA and other ribosomopathies is the activation of the tumor suppressor protein p53.^8^^,^^12^^,^^14^^,^^23^ Such an induction is thought to be caused by the inactivation of the E3 ubiquitin ligase MDM2 by free ribosomal proteins, although other mechanisms have also been proposed.^8^^,^^14^ The important role of this pathway in the pathogenesis of DBA has been demonstrated using genetic loss-of-function approaches in zebrafish, mouse, and primary human cell models.^14^^,^^24^ However, TP53-independent mechanisms must take place in parallel given that not all the DBA-like phenotypic manifestations are rescued by the elimination of TP53 in the foregoing experimental models.^24^^,^^25^^,^^26^^,^^27^^,^^28^ Consistent with this idea, it has been described that the inefficient translation of the mRNA for the master hemopoietic regulator GATA protein 1 (GATA1) is involved in the erythroid failure of patients with DBA.^29^^,^^30^^,^^31^^,^^32^ The special sensitivity of the *GATA1* transcript to ribosomal subunit deficits is attributed to its short and unstructured 5′ untranslated region (UTR), a molecular feature that confers high translation efficiencies.^32^ To date, GATA1 is the only transcription factor essential for erythropoiesis that has been linked to the development of DBA. It is worth noting, however, that such implication is under debate nowadays given that several reports have detected normal levels of GATA1-regulated gene expression programs in erythroid precursors from patients with DBA.^31^^,^^33^ The participation of other translation-sensitive transcripts (*HSP70*, *BAG1*, *CSDE1*) has been proposed as a cause for the erythroid failure as well.^34^ However, unlike the case of *GATA1*, the implication of this alternative candidates is unclear as they do not encode proteins with driving roles in erythropoiesis. Adding further support for the potential participation of other alterations in the disease, the aforementioned mechanisms do not explain specific features found in bone marrow cells from patients with DBA such as, for example, the increase in erythroid adenosine deaminase enzyme (eADA) activity, the reduction in the endogenous glucocorticoid pathway activity, or the induction of inflammatory pathways.^8^^,^^10^^,^^31^^,^^35^^,^^36^ Taken together, these data suggest that defects in ribosomal proteins or ribosome biogenesis factors might contribute to the clinical manifestations found in patients with DBA and, possibly, other ribosomopathies through the engagement of additional pathobiological programs. Further emphasizing the limited understanding of the molecular basis of these diseases, a recent report has found that the erythroid defect of DBA appears to be more severe in patients bearing mutations in a gene encoding a ribosomal protein of the small subunit (RPS) than in those carrying mutations in a gene encoding a ribosomal protein of the large subunit (RPL).^31^ Conversely, the proportion of nonhematological congenital malformations appears to be higher in patients with mutations in genes for RPLs than in genes for RPSs.^16^^,^^37^ This is quite puzzling because, prima facie, the two main mechanisms described above (p53 activation and impaired mRNA translation) should be similarly engaged by mutations targeting the 40S or the 60S biosynthetic branches.

In this work, we aimed at identifying additional programs that could be deregulated in cells undergoing ribosome biogenesis defects. To this end, we used both published RNAseq and Riboseq datasets, and wet-lab data from experimental cell conditions that mimicked the effect induced by mutations that impair the production of either the small or large ribosomal subunits. Using this approach, we have found that the impaired expression of ATF4, a pro-growth and stress-response transcription factor that plays important roles in erythropoiesis,^38^^,^^39^^,^^40^^,^^41^^,^^42^^,^^43^^,^^44^^,^^45^^,^^46^^,^^47^^,^^48^ is a p53-and cell type-independent event that is commonly associated with ribosomal protein deficiencies in many cell types. Such a reduction in ATF4 protein is achieved via two mechanisms: (i) a decrease in the levels of actively translating *ATF4* mRNAs, which is the primary defect in RPL deficiencies, and (ii) a decrease in *ATF4* mRNA stability that leads to a marked reduction in overall transcript abundance, which is the primary defect in RPS deficiencies. This latter feature provides a mechanistic explanation for the aggravated clinical phenotypes recently found in patients with DBA bearing mutations in genes encoding 40S ribosomal proteins.

## Results

### Experimental set up to identify early events associated with defects in the synthesis of 40S ribosomal subunits

**Figure 1:**
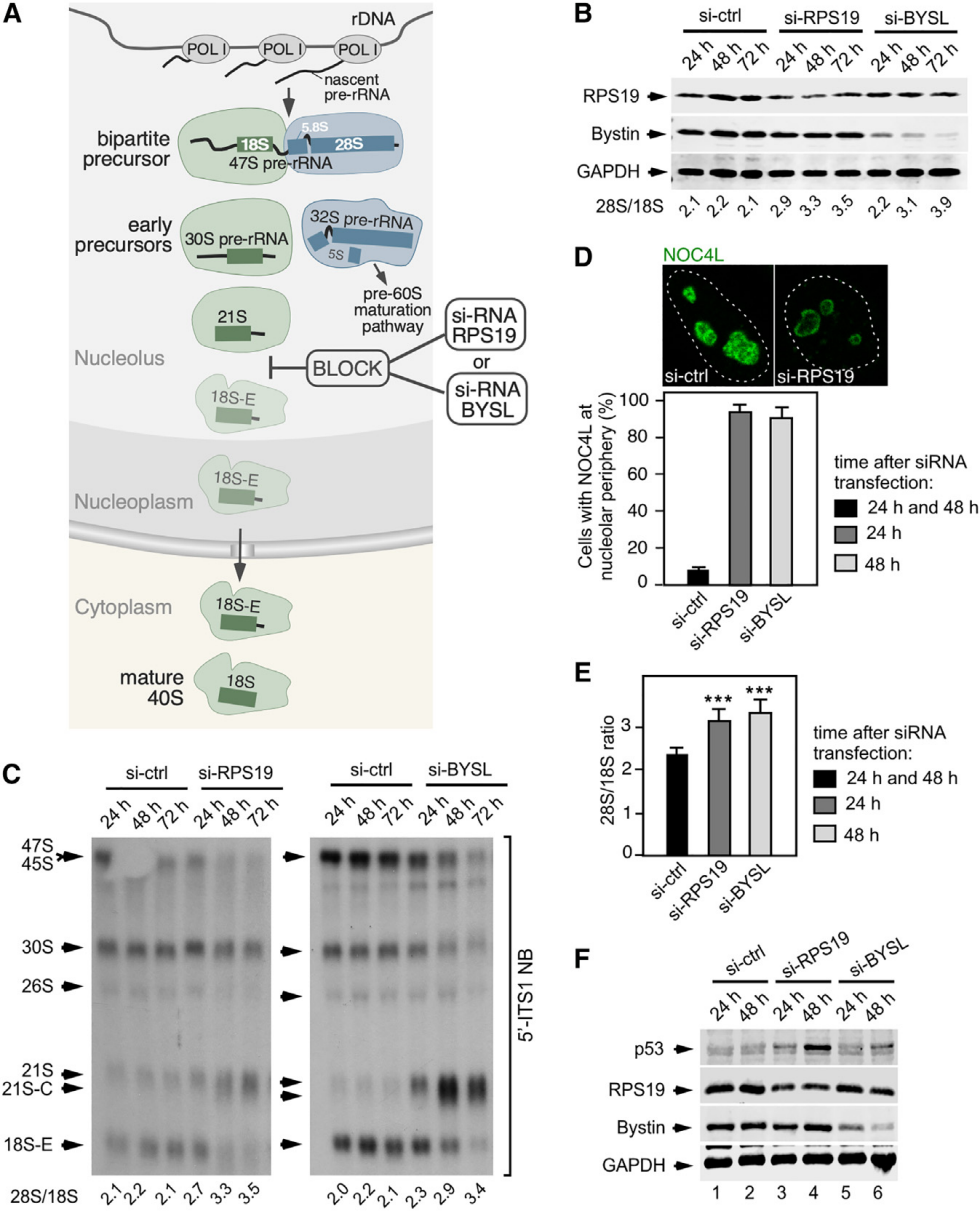
Experimental set up to analyze early events associated with defects in the synthesis of 40S ribosomal subunits (A) Cartoon showing the step of the 40S ribosomal subunit maturation pathway that is blocked by a defect in the production of either RPS19 (si-RNA RPS19) or bystin (si-RNA BYSL). For simplicity, it is represented just a subset of 40S preribosomal intermediates. Schemes of the pre-rRNA species present in each intermediate are shown. (B) Extents of protein depletion and ribosome subunit imbalance in *RPS19* and *BYSL* knockdown cells. Western blot analyses showing the levels of RPS19, bystin and the loading control (GAPDH) in total protein extracts from HCT116 cells transfected with the indicated si-RNAs and harvested 24 h, 48 h and 72 h after transfection. An aliquot of the very same transfected cells was taken for total RNA preparation to calculate the 28S/18S mature rRNA ratios (shown at the bottom of each lane) using an Agilent bionalyzer. si-ctrl, control si-RNA. (C) Accumulation of 21S/21S-C and loss of 18S-E pre-rRNAs upon knockdown of either *RPS19* (left panel) or *BYSL* (right panel). Northern blot analyses showing the abundance of different pre-rRNA species in cells treated as indicated in B. A 5′-ITS1 probe that detects all the pre-rRNA precursors of the 18S rRNA was used. Total RNAs were prepared with the Trizol method. The 28S/18S ratio of each sample is indicated at the bottom of each lane. NB, Northern blot. (D) Accumulation of the ribosome biogenesis factor NOC4L in the periphery of the nucleolus in RPS19 and bystin deficient cells. Microscopy analysis of representative cells that endogenously express the NOC4L-GFP fusion protein after 24 h of transfection with si-ctrl or si-RPS19 (top panel), and quantification of cells exhibiting NOC4L concentrated in the nucleolar periphery at the indicated time points after si-RNA transfection (bottom panel). The nucleus is indicated by the dashed line. Data are the mean ± s.d. from 40 cells of each condition in experimental duplicates. (E) Quantitation of relative ribosomal subunit abundance in cells deficient for RPS19 or bystin. Mean values of 28S/18S rRNA ratios in cells harvested at the indicated time points after siRNA transfection (*n* = 3 independent experiments). Data represent the mean ± SEM. Statistical values obtained using the unpaired two-tailed Student’s t test are given relative to si-ctrl cells. ∗∗∗, *p* ≤ 0.001. (F) Increase in the levels of p53 upon the depletion of either RPS19 or bystin. Western blot analyses showing the levels of p53, RPS19, bystin and the loading control (GAPDH) in cells transfected with the indicated si-RNAs, harvested at the indicated times after si-RNA transfection.

We hypothesized that cells might induce or repress p53-independent biological programs in response to defects in ribosome biogenesis. To assess this idea in the case of defective synthesis of 40S ribosomal subunits, we decided to analyze the impact of the RPS19 haploinsufficiency in the cell transcriptome. To this end, we resorted to a small interfering RNA (siRNA) approach to knockdown the *RPS19* mRNA in HCT116 cells, a colorectal cancer cell line suitable to identify alterations induced by ribosome biogenesis defects,^49^ study mechanisms of mRNA stability and translation regulation in ribosomal protein deficits,^50^ and dissect apart p53+ and p53-dependent processes using its *TP53* null isogenic variant.^51^ RPS19 was chosen because it is the most frequent genetic alteration (∼25% of cases) that contributes to DBA development.^22^ This ribosomal protein is essential for the maturation of the 21S pre-rRNA-containing preribosomes within the nucleolus (Figure 1A).^52^^,^^53^^,^^54^ To mimic the haploinsufficiency found in DBA, we set up knockdown conditions that led to a 50–65% reduction of RPS19 protein levels relative to those found in control cells (Figure 1B, upper panel). Such conditions induced a time-dependent partial decrease (35–40% reduction relative to the control levels) of 40S ribosomal subunits (Figure 1B, see 28S/18S rRNA ratios).

To further ensure the identification of potential effects specifically elicited by the block in 40S subunit synthesis, we performed in parallel the siRNA-mediated knockdown of *BYSL*, a transcript that encodes a ribosome biogenesis factor (bystin) that is involved in the very same 40S maturation step in which RPS19 is involved (Figure 1A).^55^ Although the loss of protein in the *BYSL* knockdown followed different kinetics when compared to the RPS19 depletion (Figure 1B, middle panel), the overall reduction in 40S subunits in the transfected cells was similar in both *BYSL* and *RPS19* knockdown conditions (Figure 1B, compare 28S/18S rRNA ratios in both cases).

As expected, the knockdown of each of those transcripts led to the accumulation of the 21S pre-rRNA species (Figure 1C) and the concentration of the 40S ribosome biogenesis factor NOC4L in the most external layer of the nucleolus (Figure 1D). The change in the intranucleolar localization of NOC4L is typically seen in cells undergoing 21S pre-rRNA maturation blockage.^56^ Importantly, the use of this microscopy-based readout also indicated that the siRNAs for *RRP19* and *BYSL* induced homogeneous phenotypes in the majority (>90%) of the transfected cells (Figure 1D). Time-course experiments showed that, to obtain moderate and comparable effects in terms of impact on 40S ribosomal subunit production, we had to culture the *RPS19* siRNA and *BYSL* siRNA transfected cells for 24 h and 48 h, respectively (Figure 1E). Cells harvested at these two time points also exhibited a similar accumulation of p53 (Figure 1F, compare lanes 1, 3, and 6). Due to this, these time-points were chosen for most of the subsequent experiments performed in this work with HCT116 cells.

Despite the detectable defects associated with the block in the maturation of the 21S pre-rRNA-containing preribosome, and the subsequent decrease in cell proliferation, (Figure S1A), we found that the *RPS19* or *BYSL* knockdown cells did not exhibit any statistically significant effect on other cell parameters such as viability or overall nucleolar morphology at the chosen post-transfection time-points (Figure S1B and data not shown). In line with this, we observed that the nucleoli of the knockdown cells also remained functional in their ability to buffer damaged proteins induced by a transient heat shock treatment (Figure S2A).^57^ We also found, using subcellular fractionation experiments with the previously reported PSE method,^58^ that the solubilization pattern of the ribosomal proteins did not significantly change upon the reductions in the amounts of RPS19 or bystin (Figure S2B). The lack of accumulation of insoluble ribosomal protein aggregates and the lack of activation of the induced stress response (ISR), inferred by the absence of the accumulation of phosphorylated eIF2α (Figure S2C), indicated that the *RPS19* and *BYSL* knockdown cells are not undergoing free ribosomal protein proteotoxic stress either. Taken together, these data indicate that under the chosen siRNA transfection conditions, the partial dampening of *RPS19* or *BYSL* transcripts does not induce a terminal or lethal phenotype that could obscure the detection of potential responses of cells at the genome-wide transcriptome level.

Finally, to further facilitate the interpretation of our gene expression analyses, we performed them using *TP53*^+/+^ and *TP53*^−/−^ isogenic variants of the HCT116 cell line in order to detect the potential engagement of p53-independent gene expression programs upon the depletion of either the *RPS19* or the *BYSL* mRNAs.

### The deficiency in RPS19 or bystin triggers p53-dependent and independent gene expression programs

**Figure 2:**
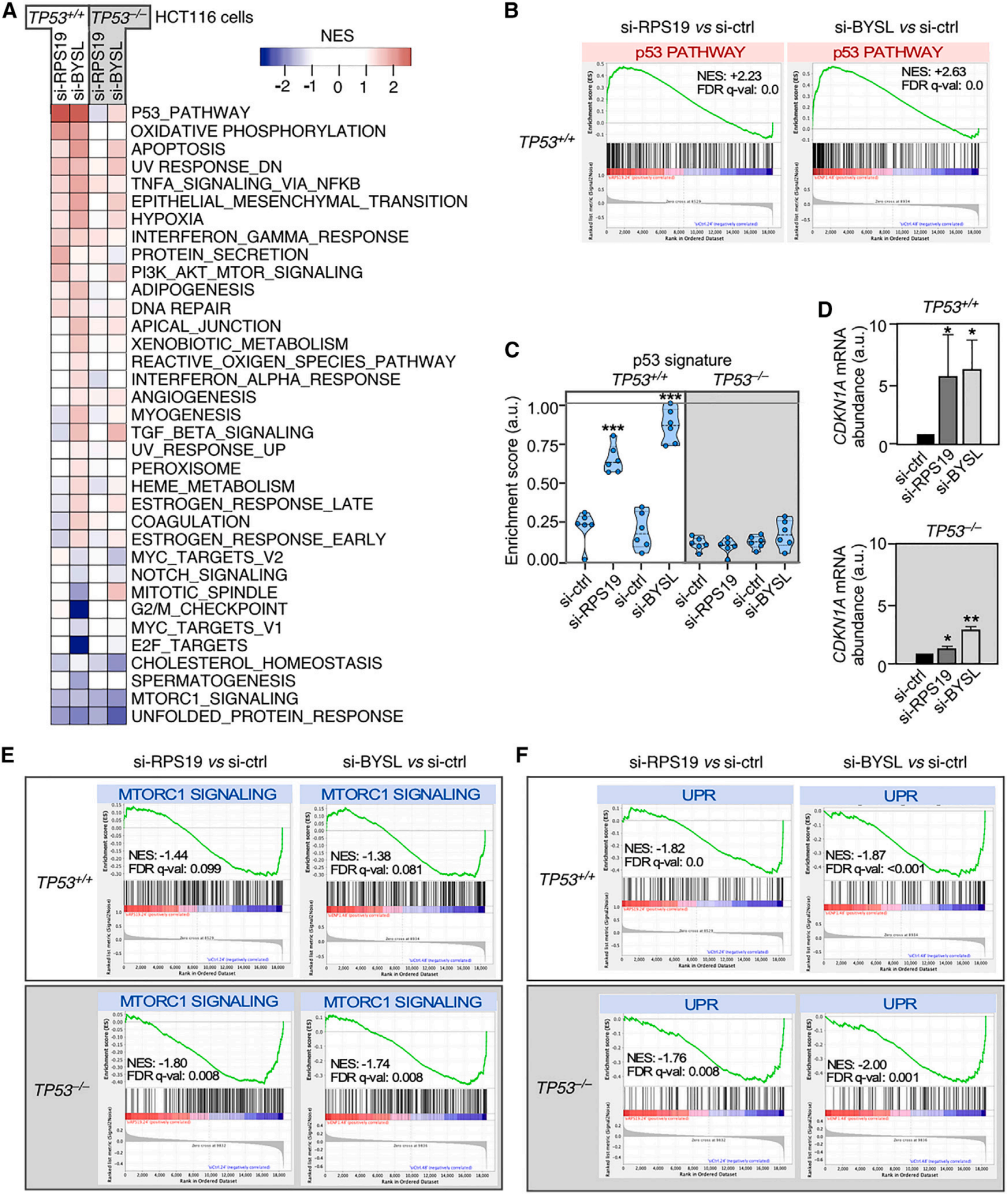
A deficiency of either RPS19 or bystin affects both p53-dependent and independent gene expression programs (A) Heatmap showing hallmark gene signatures enriched and depleted upon the knockdown of *RPS19* or *BYSL* as unveiled by GSEA of microarray gene expression data. The conditions assayed were HCT116 *TP53*^*+/+*^ and HCT116 *TP53*^*−/−*^ cells transfected with si-RNAs harvested at 24 h (si-ctrl and si-RPS19) and 48 h (si-ctrl and si-BYSL) after transfection. The heatmap includes all gene sets (35 in total) found significantly altered (FDR q value <0.25) in at least one of the four comparing conditions inspected in the analyses: si-RPS19 versus si-ctrl (24 h), si-BYSL versus si-ctrl (48 h) in both *TP53*^*+/+*^ and *TP53*^*−/−*^ cells. The color scale represents the normalized enrichment score (NES). Enriched and depleted gene sets are shown in red and blue colors, respectively. (B) Upregulation of the p53 gene signature in *TP53*^*+/+*^ cells upon knockdown of *RPS19* or *BYSL*. GSEA plots obtained from the transcriptomic data described in A showing the enrichment of the Hallmark p53 gene set upon knockdown of *RPS19* or *BYSL*. The NES and false discovery rate values (FDR, using q values) are indicated inside the GSEA graphs. (C) Relative enrichment scores of the p53 signature in all the conditions described in A. Data correspond to two independent experiments that analyzed three biological replicates of each condition. Data represent the mean ± SEM. Statistical values obtained using the unpaired two-tailed Student’s t test are given relative to si-ctrl cells. ∗∗∗, *p* ≤ 0.001 (*n* = 6 microarrays per condition). a.u., arbitrary units. (D) qRT-PCR analysis of *CDKN1A* mRNA abundance in *TP53*^+/+^ (upper graph) and *TP53*^*−/−*^ (bottom graph) cells transfected with the indicated si-RNAs. Values are normalized to GAPDH and are relative to the levels in cells transfected with the si-ctrl and harvested at the matched time-points after transfection (which was given an arbitrary value of 1). Data represent the mean ± SEM. Statistical values obtained using the unpaired two-tailed Student’s t test are given relative to si-ctrl cells. ∗, *p* ≤ 0.05; ∗∗, *p* ≤ 0.01. *n* = 3. a.u., arbitrary units. (E and F) GSEA plots obtained from the transcriptomic data described in A showing the downregulation of the Hallmark MTORC1 signaling (E) and UPR (F) gene sets upon knockdown of either *RPS19* or *BYSL* in *TP53*^*+/+*^ and *TP53*^*−/−*^ cells. The NES and FDR values are indicated as in B. UPR, unfolded protein response.

Using Affymetrix expression microarray analyses, we found that the partial loss of RPS19 triggers the differential expression of 375 and 119 genes in *TP53*^+/+^ and *TP53*^−/−^ HCT116 cells, respectively (Table S1). In the case of the *BYSL* knockdown, we detected 821 and 286 differentially expressed genes in *TP53*^+/+^ and *TP53*^−/−^ HCT116 cells, respectively (Table S1). *In silico* gene set expression analyses (GSEA) revealed a total of 35 functional gene sets that were differentially enriched in at least two of the *RPS19* and/or *BYSL* knockdown conditions (Figure 2A). Out of those, the upregulated gene sets showing the highest enrichment score in *TP53*^+/+^ cells included signatures for p53-regulated genes (Figures 2A and 2B) and for oxidative phosphorylation (Figure 2A). This latter gene program is known to be regulated by p53.^59^ Consistent with this, the foregoing gene signatures were not detected in *TP53*^−/−^ HCT116 cells (Figures 2A and 2C). These GSEA data were consistent with the initial immunoblot analysis showing that the *RPS19* and *BYSL* knockdowns promote a similar increase in the abundance of p53 protein in *TP53*^+/+^ HCT116 cells (see above, Figure 1F). Corroborating these data, we demonstrated using quantitative reverse transcription polymerase chain reaction (qRT-PCR) experiments that the expression of the p53-regulated *CDKN1A* gene was induced at similar levels in *RPS19* and *BYSL* knockdown *TP53*^+/+^ HCT116 cells (Figure 2D).

Our GSEA also revealed that the upregulated transcriptome of the knockdown cells correlates with the enrichment of gene signatures previously detected in either bone marrow cells from patients with DBA or in DBA cellular models. Those include signatures linked to several inflammation-related pathways such as those stimulated by the tumor necrosis factor α and interferon γ.^31^^,^^35^^,^^60^^,^^61^ These signatures, which show enrichment scores lower than the p53 pathway and oxidative phosphorylation gene sets described above, appear to be p53-independent in the *RPS19* but not in the *BYSL* knockdown cells (Figure 2A). In contrast, we did not detect the enrichment of the signature related to proteasome activity that had been previously observed in patients with DBA.^31^ This suggests that the induction of this gene signature is probably an adaptation or an indirect downstream event rather than a direct consequence of the impairment in ribosome biogenesis. Collectively, these results indicate that our experimental conditions can unveil conserved pathways that are triggered in a cell autonomous manner in DBA and, possibly, other ribosomopathies. They also further support the idea that one of the main responses triggered by the ribosome synthesis defects in this anemia is the upregulation of p53-dependent programs.

Our GSEA also revealed that the siRNA-mediated depletion of either *RPS19* or *BYSL* is associated with a marked downmodulation of gene signatures linked to the signaling of the mammalian target of rapamycin complex 1 (mTORC1) and the activation of the unfolded protein response (UPR) (Figures 2A, 2E, and 2F). These two gene sets become downregulated at similar levels irrespectively of the p53 functional status of HCT116 cells (Figures 2A, 2E, and 2F), thus indicating that their expression must be under the control of other transcriptional regulators. Interestingly, to the best of our knowledge, these two gene signatures have not ever been identified in previous analysis using samples from DBA or in any other ribosomopathy condition. This led us to dissect the underlying mechanism that was associated with the downregulation of these two gene expression programs.

### The RPS19 deficiency causes a reduction in the expression of ATF4-regulated genes

**Figure 3:**
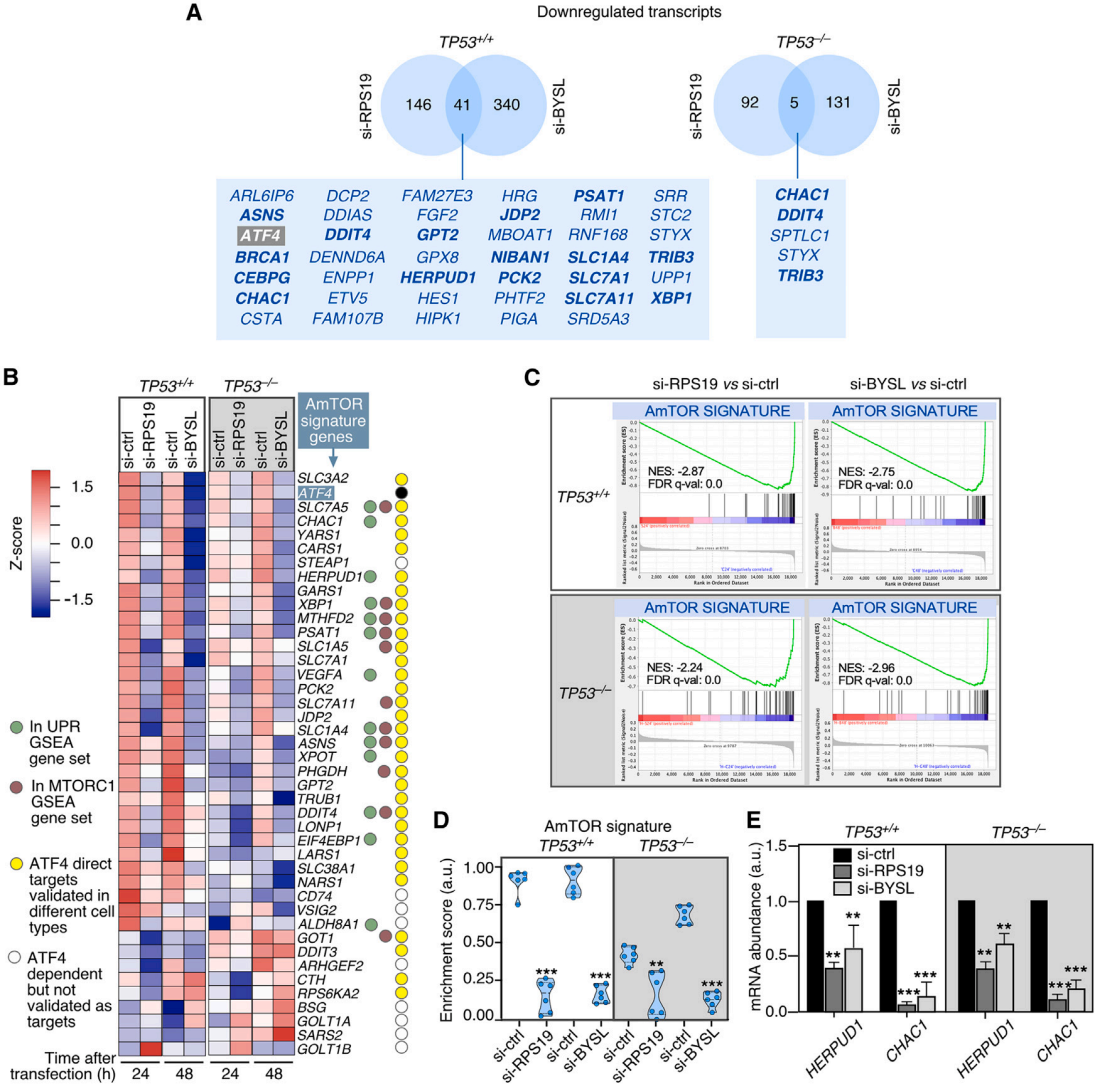
A deficiency of either RPS19 or bystin causes a loss of ATF4-regulated gene expression programs (A) Differentially expressed genes downregulated upon the knockdown of either *RPS19* or *BYSL* in HCT116 *TP53*^*+/+*^ and *TP53*^*−/−*^ cells. Venn diagrams depicting the number and overlap of transcripts downregulated in RPS19- and BYSL-deficient cells. Direct targets of ATF4 are shown in bold letters. Gene lists per category are provided in Table S1 (B) Downregulation of ATF4-dependent genes upon knockdown of either *RPS19* or *BYSL* in both *TP53*^*+/+*^ and *TP53*^*−/−*^ cells. Heatmap representing the expression of the ATF4-mTORC1 (AmTOR) signature, a group of 41 genes induced by ATF4 downstream of mTORC1 signaling in growing cells. Circles on the right indicate the genes included in the Hallmark UPR GSEA gene set (green circles), genes included in the Hallmark MTORC1 signaling GSEA gene set (brown circles), genes directly activated by ATF4 (yellow circles), and genes whose expression is dependent on ATF4 but are not directly activated by this transcription factor (white circles). Increased and decreased mRNA abundance are shown in red and blue colors, respectively. (C) GSEA plots showing the downregulation of the AmTOR signature in the four pair comparisons subject to analyses. The NES and FDR values are indicated inside the GSEA graphs. (D) Relative enrichment scores of the AmTOR signature in all the experimental conditions represented in the heatmap shown in B. Data correspond to two independent experiments that analyzed three biological replicates of each condition. Data represent the mean ± SEM. Statistical values obtained using the unpaired two-tailed Student’s t test are given relative to si-ctrl cells. ∗∗, *p* ≤ 0.01; ∗∗∗, *p* ≤ 0.001 (*n* = 6 microarrays per condition). a.u., arbitrary units. (E) Decrease in the mRNA abundance of two ATF4 target genes upon the depletion of RPS19 or bystin. qRT-PCR analysis of *HERPUD* and *CHAC1* mRNA levels in *TP53*^*+/+*^ and *TP53*^*−/−*^ cells transfected with the indicated si-RNAs and harvested at 24 h (si-ctrl, si-RPS19) and 48 h (si-ctrl, si-BYSL) after transfection. Values for each mRNA are normalized to GAPDH and are relative to the levels in cells transfected with the si-ctrl (which was given an arbitrary value of 1). Data represent the mean ± SEM. Statistical values obtained using the unpaired two-tailed Student’s t test are given relative to si-ctrl cells. ∗, *p* ≤ 0.05; ∗∗, *p* ≤ 0.01; ∗∗∗, *p* ≤ 0.001. *n* = 3. a.u., arbitrary units.

To uncover the reasons underlying the p53-independent downmodulation of the mTORC1 and UPR pathways seen in HCT116 cells, we further analyzed the differentially expressed genes commonly downregulated by the knockdowns of the *RPS19* and *BYSL* transcripts. Using this approach, we found a common set of 41 (in the case of *TP53*^+/+^ HCT116 cells; Figure 3A, left panel) and five (in the case of *TP53*^−/−^ HCT116 cells; Figure 3A, right panel) genes. Interestingly, many of these genes have been previously recognized as targets for the transcription factor ATF4 (Figure 3A, bottom, genes in bold font). Further supporting this link, we found that the levels of the *ATF4* transcript itself were reduced in both *RPS19* and *BYSL* knockdowns regardless of the TP53 status of HCT116 cells (Figure 3B, second row from top). The possible involvement of ATF4 in the downregulated gene sets was further suggested by its known function as a regulator of metabolic and redox processes downstream of mTORC1, the UPR, and the integrated stress response (ISR).^38^^,^^39^^,^^40^^,^^41^^,^^42^^,^^43^ It is worth noting that the activation of ATF4 expression in those processes is known to be regulated by either translational- (mTORC1-dependent and ISR responses) or mRNA stability-based (mTORC1-dependent responses) mechanisms.^43^^,^^62^^,^^63^^,^^64^

To explore the possibility that changes in ATF4 expression were responsible for the p53-independent gene programs downmodulated in *RPS19* and *BYSL* knockdown cells, we generated a gene signature of 41 genes whose expression is induced by ATF4 downstream of mTORC1 and phosphorylated IF2α in normal and stressed cells, respectively.^40^ This gene set is mainly composed of direct ATF4 targets (32 genes; see Figure 3B, yellow dots), half of which are present in the GSEA gene sets for the mTORC1 (11 genes; see Figure 3B, brown dots) and/or in the UPR response (13 genes; see Figure 3B, green dots). We also included *ATF4* itself (Figure 3B, black dot) plus eight additional genes whose expression is induced by mTORC1 in an ATF4-dependent manner^40^ (Figure 3B, white dots). This ATF4 gene signature includes genes involved in aminoacyl tRNA synthesis (i.e., *CARS*, *GARS*, *XPOT*), amino acid synthesis and one-carbon metabolism (i.e., *ASNS*, *PSAT1*, *GPT2*), amino acid transport (*SLC* family genes), and glutathione metabolism (*CHAC1*) that are activated downstream of mTORC1 in normally growing cells to promote protein synthesis and regulate glutathione levels.^40^ This collection of genes will be referred to hereafter as the ATF4–mTORC1 (AmTOR) signature. Using our microarray expression data, we found that the mRNAs for ATF4 and the majority of AmTOR signature components displayed a similar downregulation in both *TP53*^+/+^ and *TP53*^−/−^ HCT116 cells upon the depletion of either the *RPS19* or the *BYSL* transcript (Figures 3B–3D). We validated the downregulation of two of those *ATF4* direct targets (*HERPUD1*, *CHAC1*) using qRT-PCR analyses (Figure 3E). These results indicate that the block of the 40S subunit synthesis pathway provoked by an RPS19 deficiency leads to a decrease in the expression of genes regulated by ATF4 in normally growing cells.

### Distinctive alterations of ATF4 expression in RPS19- and RPL5-deficient cells

**Figure 4:**
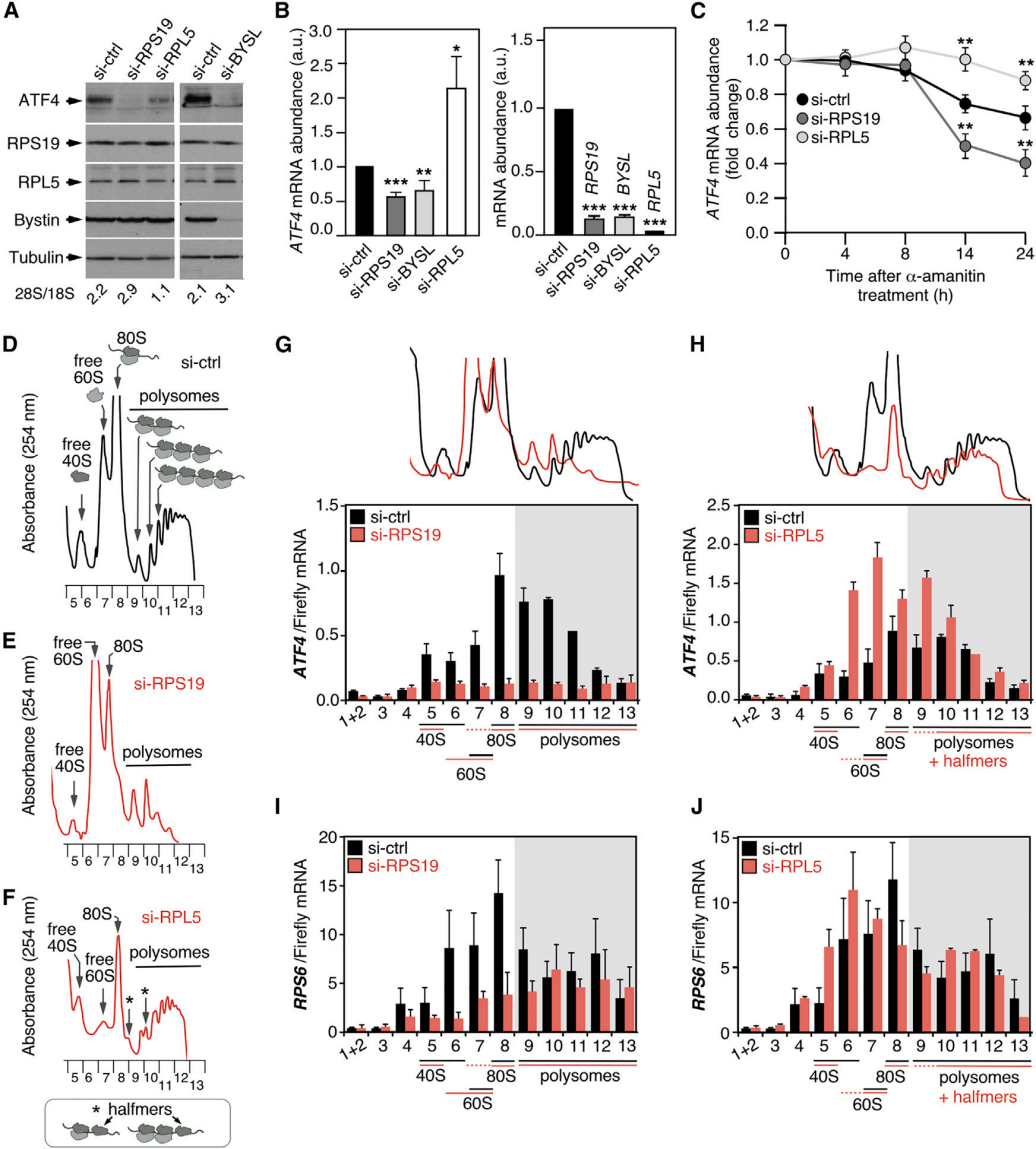
Distinctive alterations of ATF4 expression in RPS19- and RPL5-deficient cells (A) Decrease in ATF4 protein upon knockdown of either *RPS19*, *BYSL* or *RPL5*. Western blot analyses showing the levels of ATF4, RPS19, bystin and the loading control (tubulin) in total protein extracts from HCT116 cells transfected with the indicated si-RNAs and harvested 24 h (si-ctrl, si-RPS19 and si-RPL5) or 48 h (si-ctrl and si-BYSL). An aliquot of the very same transfected cells was taken for total RNA preparation to calculate the 28S/18S mature rRNA ratios (shown at the bottom of each lane) using an Agilent bionalyzer. (B) Altered levels of *ATF4* mRNA upon knockdown of either *RPS19*, *BYSL* or *RPL5*. qRT-PCR analysis of *ATF4* mRNA (panel on the left) and silenced transcript (panel on the right) abundances in HCT116 cells transfected with the indicated si-RNAs. Values for each mRNA are normalized to GAPDH and are relative to the levels in cells transfected with the time-matched si-ctrl. Data represent the mean ± SEM. Statistical values obtained using the unpaired two-tailed Student’s t test are given relative to si-ctrl cells. ∗, *p* ≤ 0.05; ∗∗, *p* ≤ 0.01; ∗∗∗, *p* ≤ 0.001. *n* = 3. a.u., arbitrary units. (C) The loss of RPS19 causes a decrease of *ATF4* mRNA stability, and the loss of RPL5 causes an increase of *ATF4* mRNA stability. Transcription was inhibited with α-amatinin immediately after si-RNA transfection and the levels of *ATF4* transcript were measured at the indicated time points thereafter by qRT-PCR. Values are normalized to GAPDH and are relative to the values of cells just before si-RNA transfection (time 0). Data represent the mean ± SEM. Statistical values obtained using the unpaired two-tailed Student’s t test are given relative to si-ctrl cells. ∗, *p* ≤ 0.05; ∗∗, *p* ≤ 0.01; *n* = 3. (D–F) Representative polysome profiles of HCT116 cells transfected with si-ctrl, si-RPS19 and si-RPL5, harvested at 24 h after transfection, and subjected to sucrose gradient sedimentation analyses. Asterisks indicate the peaks corresponding to mRNAs bound by one ribosome and a halfmer, and mRNAs bound by two ribosomes and one halfmer. (G–J) Distribution of the *ATF4* (upper panels) and *RPS6* (bottom panels) mRNAs across sucrose gradient fractions in si-ctrl- and si-RPS19- transfected cells (left panels); and in si-ctrl and si-RPL5-transfected cells (right panels). The mRNA levels in each fraction were quantitated by qRT-PCR using the amount of the spike Firefly mRNA for normalization, as described in STAR Methods. The fractions containing 40S, 60S, 80S and polysomes are indicated at the bottom of each bar graph.

We addressed the impact of the RPS19 deficiency on ATF4 expression using western blot and RT-qPCR analyses. Consistent with the marked reductions observed in the expression of the AmTOR signature (see above Figure 3D), we found a strong decrease in ATF4 protein upon knockdown of either the *RPS19* or *BYSL* transcript in *TP53*^+/+^ HCT116 cells (Figure 4A, upper panel). Interestingly, we confirmed the results of the microarray analyses and found that the levels of *ATF4* transcript were also diminished in both si-RNA scenarios (Figure 4B; see above Figure 3B, second row from top), indicating a mechanism of downregulation at the mRNA transcription and/or stability level. As a control, we analyzed the effect of the knockdown of a 60S ribosomal protein (*RPL5*) in ATF4 levels. Consistent with its known role in 60S synthesis, the depletion of RPL5 was associated with the reduction of 60S ribosome subunits in cells (Figure 4A, see 28S/18S ratios). We found that the depletion of RPL5 promoted a milder reduction of ATF4 protein than that found in either the *RPS19* or the *BYSL* knockdown cells (Figure 4A, upper panel). In contrast, it led to an increase rather than to a decrease in the levels of *ATF4* mRNA (Figure 4B). The opposite effects on the abundance of *ATF4* mRNA indicated that the mechanisms downregulating *ATF4* expression in the RPS19 and RPL5 deficiencies must be different. To analyze if those differences were related to effects on transcript stability, we monitored the levels of *ATF4* mRNA under conditions of transcription inhibition with α-amanitin. We found that the stability of the *ATF4* mRNA is decreased upon the depletion of RPS19 and, in contrast, it is increased upon the depletion of RPL5 (Figure 4C). These results indicate that one mechanism underlying the loss of ATF4 protein in RPS19-deficient cells is the increase in the rate of degradation of the *ATF4* transcript. They also indicate that the mechanism in RPL5-deficient cells must be a reduction in the rate of mRNA translation because, in this case, the *ATF4* transcript is more stable and gets accumulated but the levels of protein are lower than in control cells.

To analyze changes in *ATF4* mRNA translation, we used sucrose gradient fractionation analyses that assessed the distribution of *ATF4* transcript in polysome and sub-polysome fractions. Polysome fractions contain actively translating mRNA complexes. Sub-polysome fractions contain mRNAs that might be (i) forming translation-unproductive complexes with no ribosomes bound, (ii) subject to scanning by 40S subunits but have no ribosomes bound, or (iii) initiating translation or blocked at translation initiation with one ribosome bound. As shown in the polysome profile plots, the RPS19 and RPL5 knockdowns induced the expected ribosome synthesis defects, with a loss of free 40S subunits, increase of free 60S subunits and diminished polysomes in the case of the RPS19 knockdown (compare plots in Figures 4D and 4E); and with a loss of free 60S ribosomal subunits, increase of free 40S ribosomal subunits, decrease of polysomes and increase of halfmers in the case of the RPL5 knockdown (compare plots in Figures 4D and 4F). Consistent with the quantifications of total mRNA levels (Figure 4B), RPS19-depleted cells exhibited a major reduction of the *ATF4* transcript levels in the fractions of the sucrose gradient (Figure 4G, overall transcript abundance is the sum of abundance in all 13 fractions). In strong contrast, RPL5-depleted cells exhibited an overall increase in the abundance of *ATF4* mRNA that is abnormally concentrated in sub-polysomal (40S-80S) and disome or halfmer-containing fractions (Figure 4H, compare mRNA abundance in fractions 6–9 in si-ctrl and si-RPL5 conditions), indicating an enrichment in complexes that are not being actively translated and in complexes with low ribosome occupancy. The different behaviors of the *ATF4* mRNA in RPS19- and RPL5-deficient cells resemble those previously described for 5′-TOP mRNAs, a distinct class of transcripts whose stability and silencing into translationally inactive complexes involves the formation of a complex with a 40S ribosomal subunit and a regulatory factor (LARP1).^50^^,^^65^ Indeed, we observed that the overall fractionation patterns of the *RPS6* mRNA, a 5′-TOP transcript, were similar to those seen for the *ATF4* mRNA (Figures 4I and 4J), although some noticeable differences could be observed: the extent of the loss of the *RPS6* mRNA in the RPS19 deficiency was less pronounced than the one of the *ATF4* mRNA, and the sizes of the enriched sub-polysomal complexes in the RPL5 deficiency were lower (40S-60S) in the case of the *RPS6* transcript in comparison to those of the *ATF4* transcript (40S-80S). Together, these results showed that a defective production of RPS19 compromises the stability of *ATF4* mRNA, and that a defective production of RPL5 leads to the anomalous accumulation of this transcript in translationally inactive complexes. They also indicate that the behavior of the *ATF4* mRNA in ribosomal protein deficiencies resembles the behavior of transcripts whose stability and balance between translationally active and translationally inactive depends on the levels of free 40S subunits.

### Reduction of ATF4 expression is more severe in RPS-deficient cells

**Figure 5:**
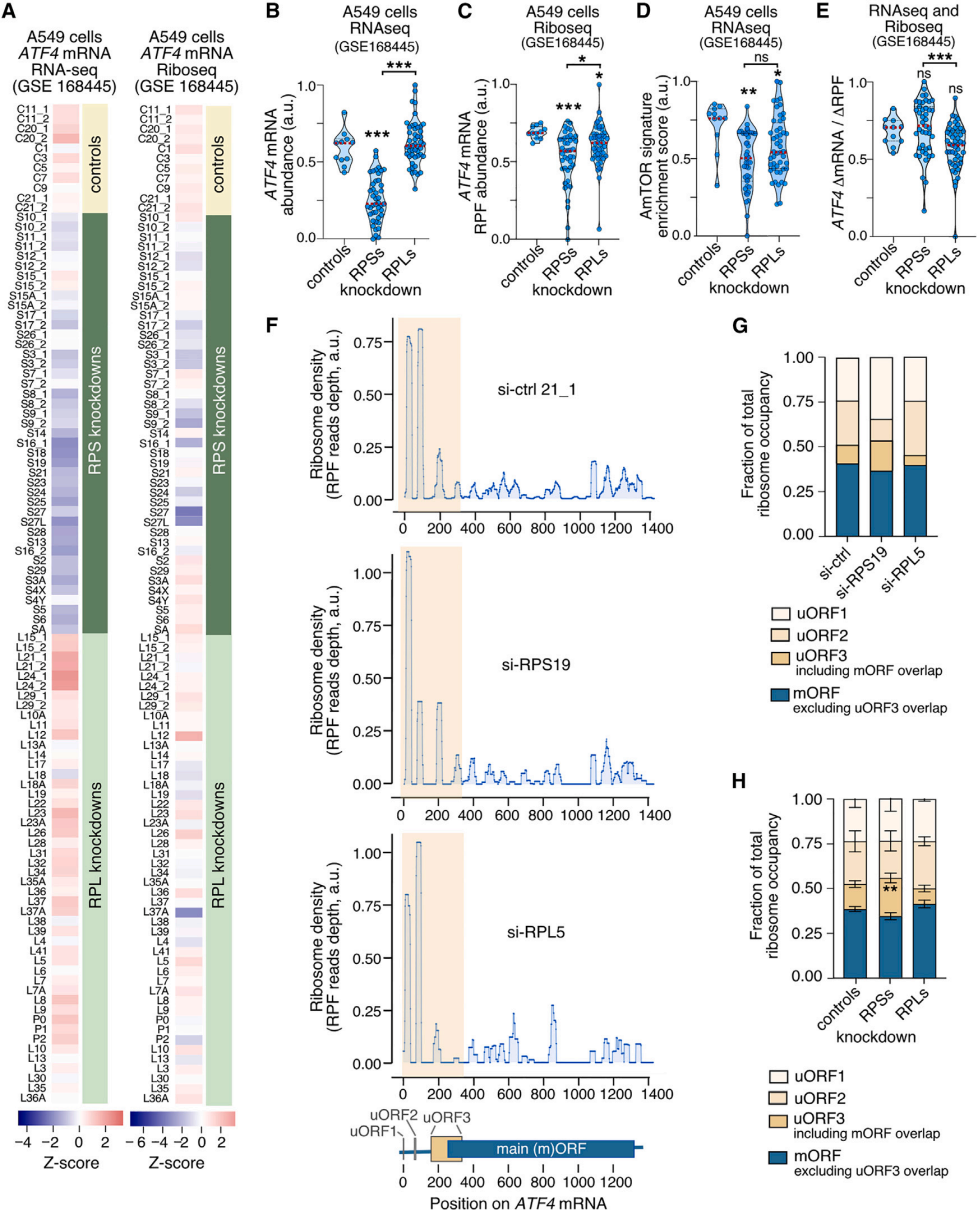
The loss of ATF4 is a common feature of ribosomal protein deficiencies, but it is more severe in RPS deficiencies (A) Relative abundance of *ATF4* mRNA (left panel) and *ATF4* mRNA ribosome-protected-fragment (RPF) abundance (right panel) in A549 cells after the individual si-RNA-mediated knockdown of 75 ribosomal proteins (31 RPSs and 44 RPLs).^66^ The values of *ATF4* and AmTOR signature mRNAs levels were retrieved from the GSE168445 RNA-seq dataset and subjected to unsupervised hierarchical clustering. The full resultant heatmap is shown in Figure S2. For simplicity, here it is shown only the heatmap row corresponding to ATF4 mRNA abundance. Relative differences in mRNA abundance are represented according to the *Z* score color scale shown at the bottom. (B–E) Dot plots comparing the *ATF4* mRNA levels (A), ATF4 mRNA ribosome-protected-fragment (RPF) abundance (B), AmTOR signature enrichment (C), and the ratio of mRNA abundance fold-change relative to the RPF abundance fold-change (D) in the 75 individual ribosomal protein knockdowns in A549 cells analyzed in A, grouped in the three indicated knockdown classes: controls, si-ctrl samples; RPSs, si-RPS samples; RPLs, si-RPL samples. Data was obtained from the GSE168445 RNA-seq (A, B, D) and Ribo-seq (C, E) dataset. Dots represent the values in individual ribosomal protein knockdowns. RPF, ribosome protected fragment. In the four plots, the red dashed lines represent the mean values. Data represent mean ± SEM. ∗, *p* ≤ 0.05; ∗∗, *p* ≤ 0.01; ∗∗∗, *p* ≤ 0.001 (one-way ANOVA and Dunnett’s multiple comparisons test (B), Kruskal-Wallis and Dunn’s multiple comparisons test (C, D), *n* = 11, 43, and 48 samples for control, RPS, and RPL knockdowns). (F) Examples of ribosome occupancies on the *ATF4* transcript in A549 cells after the knockdown of one RPS (si-RPS19) and one RPL (si-RPL5). A control sample (si-RNA C21_1) is also shown for comparison. RPF read counts were obtained from the GSE168445 Ribo-seq dataset and assigned to their corresponding positions on the *ATF4* mRNA. The diagram at the bottom indicates the positions of the three uORFs and the main (m)ORF on the *ATF4* transcript. The shaded region in each graph corresponds to the 5′-UTR plus the 5′-region of the mORF that overlaps with uORF3. (G) Relative ribosome occupancies at four consecutive regions of the ATF4 transcript in the three cases shown in F. The four regions contain uORF1, uORF2, uORF3 (including the overlap with the mORF) and mORF (excluding the overlap with uORF3). Region extensions and coordinates are shown in Figure S2A. (H) Relative ribosome occupancies at the four regions of the ATF4 transcript in control, RPS and RPL knockdowns. The graph represents the mean and SEM of values from four individual knockdowns of each class (individual plots and fraction quantitations are shown in Figure S2). ∗∗, *p* ≤ 0.01 (unpaired two-tailed Student’s t test, *n* = 4 samples per condition).

If the *ATF4* mRNA properties and functionality depended on the availability of free 40S subunits, all ribosomal protein deficiencies that decrease or increase the abundance of 40S subunits should affect *ATF4* expression in the same manner as in the RPS19 deficiency and RPL5 deficiency, respectively. To investigate *ATF4* expression in other ribosomal protein deficiencies and cell types, we used available RNA-seq and Ribo-seq data from the *TP53*^+/+^ A549 lung cancer line in which the transcripts for 44 RPLs and 31 RPSs were individually knocked down using a siRNA-mediated approach.^66^ Consistent with the results reported in the original study^66^ and our own results with HCT116 cells, the most consistent effect found in the majority of the knockdown samples (about 75% of the cases) was the activation of the p53 pathway (data not shown). Such a feature was found irrespectively of whether the knockdown transcripts encoded RPSs or RPLs (data not shown). Unlike this case, we found a clear segregation of the knockdowns for *RPS* transcripts (lower *ATF4* mRNA levels relative to controls) and for *RPL* mRNAs (similar or higher *ATF4* mRNA levels relative to controls) when the *ATF4* mRNA was interrogated (Figures 5A left panel, and 5B). This effect was similar to the one previously found in HCT116 cells depleted of RPS19 or RPL5 (see above, Figure 4B). In further agreement with the effects at the protein level found in HCT116 cells, the measurements of ribosome protected footprints (RPFs) in the Ribo-seq data from A549 cells also indicated that the overall translation of the *ATF4* mRNA was reduced in the deficiencies for proteins of both ribosomal subunits, although the effect was accentuated in the case of the *RPS* knockdown cells (Figures 5A right panel, and 5C). Consistent with this, the AmTOR gene signature also became more downregulated in RPS- than in RPL-depleted A549 cells (Figure 5D). Quantifications of RPF abundance normalized over mRNA abundance indicated that the occupancy of the *ATF4* mRNA by ribosomes was higher in *RPS* than in *RPL* knockdowns (Figure 5E). This result is consistent with the distribution in polysome and subpolysome complexes seen for the *ATF4* transcript in the *RPS19* and *RPL5* knockdowns in HCT116 cells (Figures 4G and 4H), further supporting the idea that the decrease in *ATF4* expression is mainly due to the reduction of transcript levels in the case of a deficit of 40S subunits, and to the inefficient translation of the transcript in the case of a deficit of 60S subunits. Furthermore, it indicates that the more severe defect of *ATF4* expression in *RPS* knockdown cells in HCT116 and A549 cells is due to the fact that the decrease in *ATF4* mRNA levels has a stronger impact than the decrease in translation efficiency impinged by *RPL* knockdowns.

Given that the translation efficiency of the *ATF4* mRNA is regulated by three consecutive upstream open reading frames (uORFs) (see scheme in Figure S4A),^62^^,^^63^^,^^67^ we speculated that both the diminished ribosome occupancy found in *RPL* knockdowns in A549 cells and the enrichment of translationally inefficient *ATF4* mRNAs upon *RPL5* knockdown in HCT116 cells (Figure 4H), could be the result of the preferential use of the *ATF4* uORFs rather than of the main *ATF4* ORF. To evaluate this possibility, we performed footprint assignments on the *ATF4* transcript using the Ribo-seq data from siRNA-transfected A549 cells. We could only find a mild elevation in the translation of uORF3 in the *RPS* knockdown cells when compared to *RPL* knockdown and control cells (Figures 5F and 5G, si-ctrl, si-RPS19 and si-RPL5; Figures 5H and S4, compiled data from four siRNAs of each class). This result indicates that the reduction of ATF4 protein levels in *RPL* knockdown cells is probably the consequence of a generic decrease in *ATF4* mRNA translation caused by the abnormally high levels of free 40S subunits and low levels of 60S subunits rather than by a shift in the preferential use of the *ATF4* uORFs by the remaining ribosomes.

### ATF4 expression is compromised in erythroid precursors with Diamond-Blackfan anemia-like ribosomal protein deficiencies

**Figure 6:**
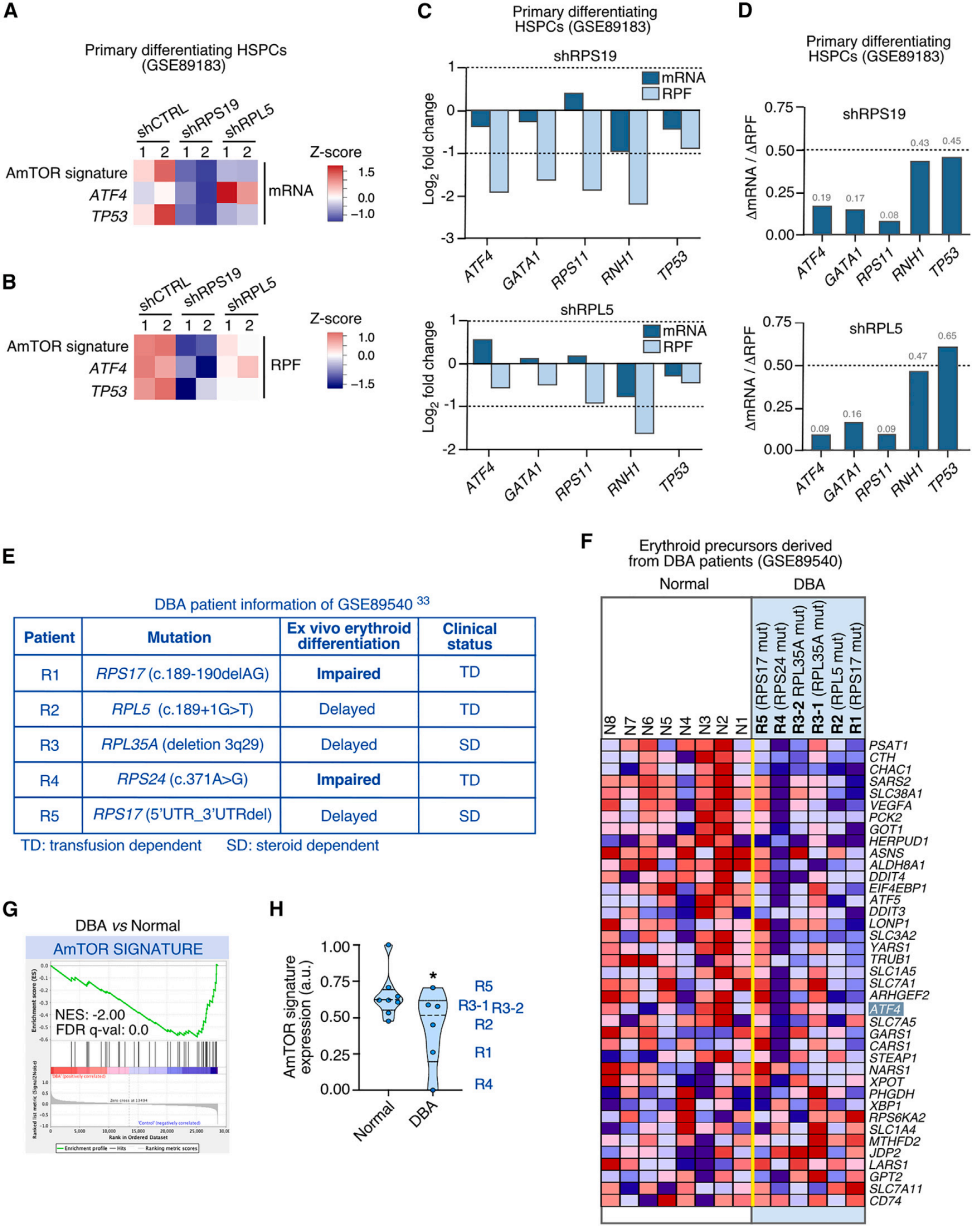
ATF4 expression is compromised in erythroid precursors with DBA-associated ribosomal protein deficiencies (A and B) Relative abundance of AmTOR signature mRNAs and ATF4 mRNA (A) and RPFs (B) in primary differentiating human HSPCs after treatment with control (shCTRL) vector or shRNAs for RPS19 or RPL5.^32^ The *TP53* transcript is included for comparison with an mRNA not regulated at the translational level. Transcript and RPF values were retrieved from the GSE89183 RNA-seq and Ribo-seq datasets, respectively. GSE89183 includes data from biological replicates (rows 1 and 2) of the RPS19 and RPL5 knockdowns. Relative differences in abundance are represented according to the *Z* score color scales shown on the right. (C) Relative changes in mRNA and RPF abundances of the indicated transcripts extracted from the same dataset used to generate the heatmaps shown in A and B. Each bar value is the average of the two values from the corresponding biological replicates. (D) Reductions in mRNA abundance relative to the reduction in RPF abundance for a selected set of transcripts. The values represented in the graphs in C were used to calculate the ratios of mRNA abundance fold-change relative to the RPF abundance fold-change. (E) Information about the patients with DBA analyzed in a previous study that performed microarray gene expression analyses (GSE89540) on erythroid precursors (CD41^–^/CD44^+^/CD235^–^) from 5 patients with DBA and 8 normal individuals obtained by the expansion and differentiation of CD34^+^ progenitors *ex vivo*.^33^ The DBA genotypes of the 5 patients are indicated. The erythroid differentiation capacity, as measured by the expression of CD235 by day 14 of culture in differentiating media, was delayed in three of the patients and impaired (the proportion of CD235^+^ cells was highly reduced) in two of the patients. (F–H) Downregulation of the AmTORC1 signature in erythroid precursors of patients with DBA. Heatmap (F), plot (G) and relative enrichment (H) of GSEA performed on the microarray data (GSE89540) of CD41^–^/CD44^+^/CD235^–^ cells from the indicated normal individuals and patients with DBA against the AmTOR signature. ∗, *p* ≤ 0.05 (unpaired one-tailed Student’s t test, *n* = 8 and 6 control and DBA samples, respectively).

Given that ATF4 plays important functions in erythroid cell homeostasis and differentiation,^44^^,^^45^^,^^47^ we hypothesized that its downmodulation could be associated with the erythroid failure found in DBA and other ribosomopathies. To explore this possibility, we investigated the expression of the AmTOR gene signature in RNA-seq and Ribo-seq datasets previously obtained from primary human hematopoietic stem and progenitor cells (HSPCs) undergoing erythroid differentiation in which the *RPS19* or *RPL5* transcripts were knocked down using a short hairpin RNA (shRNA) approach.^32^ This approach allowed the previous identification of the GATA1-and ribosomal protein-encoding transcripts as the mRNAs whose translation was most sensitive to the ribosomal protein defects found in patients with DBA.^32^ Importantly, the AmTOR gene signature only shares a minimal overlap (7.9%) with GATA1 target genes according to the ChIP-Atlas database,^68^ thus indicating that the former signature can be used to unambiguously interrogate the functional status of the ATF4 pathway in the erythroid lineage. Consistent with our previous observations in HCT116 and A549 cells, *in silico* analyses of the HSPC RNA-seq dataset showed that the expression of the AmTOR gene signature was downregulated at high and moderate levels in the *RPS19* and *RPL5* knockdown HSPCs, respectively (Figure 6A, top row). This correlated, as in the case of both HCT116 and A549 cells, with the concomitant downregulation (in the case of *RPS19* knockdown cells) and the upregulation of the *ATF4* mRNA (in the case of the *RPL5* knockdowns cells) in each of those two experimental conditions (Figure 6A, middle row). The Ribo-seq data also revealed that the overall translation of the AmTOR signature (Figure 6B, top row) and of the *ATF4* mRNA itself (Figure 6B, middle row) was also more reduced in *RPS19* than in *RPL5* knockdown HSPCs. When using *TP53* as a control for a transcript with low sensitivity to ribosome losses, we found that it exhibited similar reductions in terms of mRNA levels and overall translation (Figures 6A and 6B, bottom rows). The reductions in overall translation (fold-change in RPF abundance) of the *ATF4* mRNA were comparable to those found for three transcripts (*GATA1*, *RPS11*, *RNH1*) previously described as mostly affected at the translational level in RPS19- and RPL5-deficient HSPCs^32^ (Figure 6C). The levels of ribosomes bound to the *ATF4* mRNA were diminished both in *RPS19* and *RPL5* knockdown HSPCs (Figure 6D), indicating that upon the knockdown conditions used in these experiments with HSPCs (incubations with shRNAs for 5 days), the cells undergoing the *RPS19* deficiency not only show a decrease in the levels of *ATF4* transcript but also a decrease in translation efficiency. Altogether, these results further indicate that the impairment of *ATF4* expression is a primary and general defect triggered in cells bearing defects in ribosomal protein production, including the erythroid progenitor cells that are specifically defective in patients with DBA. In addition, they show that the sensitivity of the *ATF4* expression to reductions in 40S and 60S ribosomal subunits is comparable, or even slightly higher, to the one exhibited by the *GATA1* mRNA. These findings also indicate that, as in other cell types, the erythroid progenitors exhibit a more severe reduction in *ATF4* expression when carrying mutations in *RPS* genes than in *RPL* genes.

To further buttress the foregoing observations, we carried out *in silico* analysis using data from erythroid precursors directly isolated from patients with DBA.^33^ It is worth noting that such data are scarce and heterogeneous due to the low epidemiological incidence of the disease, the variegated nature of the disease penetrance among patients, the difficulty in obtaining primary erythroid precursors from patients, and the diversity in treatment status.^29^^,^^31^^,^^33^ We selected for our study a genome-wide gene expression dataset from erythroid precursors that were expanded and differentiated *ex vivo* from CD34^+^ progenitors isolated from five patients with DBA and eight healthy individuals.^33^ This dataset is particularly useful because it contains information about patient clinical data and the differentiation capacity of the erythroid precursors isolated in each case. The five DBA cases analyzed in the dataset include heterozygous mutations in the *RPS17* (patients R1 and R5), *RPL5* (patient R2), *RPL35A* (patient R3, analyzed in replicates), and *RPS24* (patient R4) genes (Figure 6E). These cases were associated with either defective (patients R1 and R4) or delayed (rest of patients) erythroid differentiation capacities (Figure 6E). They included transfusion dependent (three cases) and steroid dependent (two cases) patients. Despite the high variation in gene expression patterns in the control samples included in this dataset (Figure 6F), we found that the expression of 20 out of the 38 genes (52.63%) included in the AmTOR signature was reduced in the mutant erythroid precursors when compared to control cells (Figures 6F and 6G). The AmTOR gene signature was also significantly downregulated in the mutant cells (Figure 6H). Interestingly, a close inspection of these data revealed that the AmTOR signature was less affected in the cells from steroid-dependent patients (R3 and R5) that exhibit relatively high levels of *ATF4* mRNA (Figures 6H and 6F), and that the signature underwent the largest downregulation in patients (R1 and R4) that bear mutations in two *RPS* genes (Figure 6H). The latter patients displayed the most severe differentiation defects of all the patient cohort analyzed (Figure 6E). These results suggest that the function of ATF4 is severely compromised in erythroid progenitors from patients with DBA, namely those bearing *RPS* gene mutations associated with strong erythroid differentiation defects.

### Ribosomal protein deficiencies compromise the ATF4-mediated block of fetal hemoglobin expression in erythroid cells

**Figure 7:**
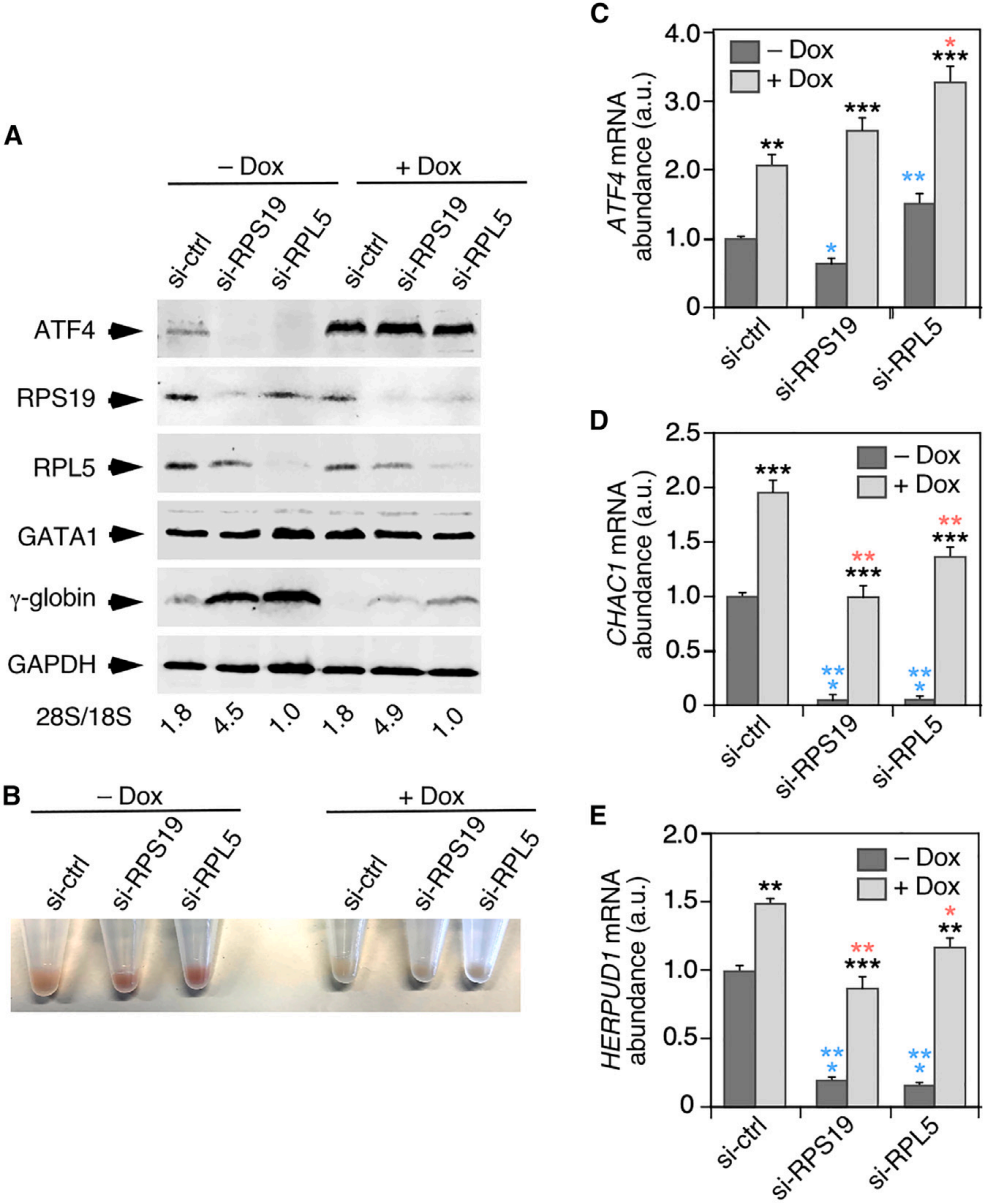
Ribosome protein deficiencies compromise the ATF4-mediated block of fetal hemoglobin expression in erythroid cells Western blot analyses of total cellular extracts (A) and cell pellets (B) from K562 cells containing a stably integrated doxycycline-inducible *ATF4* transgene that were either non-treated (-Dox) or treated (+Dox) with doxycycline for 24 h, and then transfected with the indicated si-RNAs and harvested 72 h after transfection. An aliquot of the very same transfected cells was taken for total RNA preparation to determine the 28S/18S mature rRNA ratios (shown at the bottom of each lane) using an Agilent bionalyzer. (C–E) qRT-PCR analysis of *ATF4* (C), *HERPUD* (D) and *CHAC1* (E) mRNA abundance in the cells and treatments described in A. Values for each mRNA are normalized to GAPDH and are relative to the levels in cells non-treated with doxycycline and transfected with the si-ctrl (which was given an arbitrary value of 1). Data represent the mean ± SEM. Statistical values were obtained using the unpaired two-tailed Student’s t test. *p* values are given relative to non-treated (blue asterisks) and treated (red asterisks) cells transfected with si-ctrl. We also included *p* values for the values exhibited by each si-RNA relative to those obtained in non-treated condition (black asterisks). ∗, *p* ≤ 0.05; ∗∗, *p* ≤ 0.01; ∗∗∗, *p* ≤ 0.001. *n* = 3. a.u., arbitrary units.

Finally, we investigated whether the reduction of ATF4 plays roles in cellular alterations known to be elicited by dysfunctions of RPS19 or RPL5 in erythroid cells. Most particularly, we focused on the elevated fetal γ-globin levels. Although this feature is found in both patients with RPS-DBA and RPL-DBA, recent data indicate that it is present in higher percentages of erythroid progenitors in RPS-DBA than in patients with RPL-DBA.^31^ Moreover, it is known that ATF4 represses the expression of fetal γ-globin genes in erythroid precursors.^48^^,^^69^ To this end, we knocked down the *RPS19* or the *RPL5* transcripts using a siRNA approach in the *TP53*^−/−^ erythroleukemia cell line K562 that was modified to ectopically express ATF4 upon the addition of doxycycline to the culture medium (Figure 7A, top panel). As a control, we used the similar cell line transfected with a scramble siRNA. It must be noted that the timing of the analyses in K562 cells (72 h upon siRNA transfection) implies a lengthier loss of ribosomes than in the knockdown assays performed in HCT116 and A549 cells (24 h upon siRNA transfection). We found that under these experimental conditions, the siRNAs for *RPS19* and *RPL5* produce drastic losses of the appropriate encoded proteins and of ribosomes (Figure 7A, second and third panels from top; see also 28S/18S ratios at the bottom). Despite such drastic depletions, a functional pool of ribosomes was still present in the knockdown cells as inferred from the fact that ATF4 could still be effectively overexpressed upon the addition of doxycycline to the cells (Figure 7A, top panel). The amounts of ribosomes were also sufficient to maintain the steady-state levels of GATA protein, as indicated by the comparable western blot signals in control and siRNA-transfected cells not treated with doxycycline (Figure 7A, fourth panel from top). In contrast, we found that both the *RPS19* and *RPL5* knockdown conditions induced major losses of both endogenous ATF4 protein (Figure 7A, top panel) and ATF4 target genes such as *CHAC1* (Figure 7D) or *HERPUD1* (Figure 7E). We found no signs of ISR activation in the si-ctrl condition (Figure S5), indicating that the changes in ATF4 levels are not caused by a downregulation of the ISR pathway induced by the *RPS19* and *RPL5* knockdowns. Such a downregulation has been previously seen in the case of primary cultures of erythroid cells and found to be caused by a protein/heme imbalance.^70^^,^^71^ As expected, the expression of the ATF4 target genes was restored upon the doxycycline-mediated expression of ATF4 (Figures 7D and 7E). Strikingly, we observed that the depletion of RPS19 or RPL5 induced the expression of the fetal γ-globin in K562 cells (Figures 7A, fifth panel from top; and 7B). This effect was blocked by the doxycycline-induced expression of ATF4 in cells (Figures 7A, fifth panel from top; and 7B). Collectively, these results further confirm that *ATF4* mRNA translation is highly sensitive to the loss of ribosomal subunits in all cell types analyzed. They also indicate that the downregulation of ATF4 is associated with the abnormal upregulation of fetal hemoglobin that is typically found in erythroid precursors from patients with DBA. In contrast, our data suggest that GATA1 must not be a direct player in this process.

## Discussion

In this work, we aimed at identifying cell type- and p53-independent biological programs that could be associated with specific clinical defects found in ribosomopathies and, most particularly, in DBA. To avoid being confused by indirect effects caused by a severe depletion of ribosomal subunits, we selected experimental conditions that only caused moderate disturbances in the intracellular ratios of 40S and 60S ribosomal subunits. Furthermore, we analyzed in parallel the deficits of RPS19 and bystin to identify the changes derived from the 40S subunit maturation defect and not by any other unrelated defect. Subsequently, we performed comparative analyses of deficits in RPSs and RPLs to determine if those changes are 40S-dependent or a more general response to ribosome subunit depletion. Finally, we collected data from HSPCs undergoing erythroid differentiation and from erythroid precursors directly obtained from patients with DBA to extrapolate our observations with cultured cells to more physiologically relevant settings. This experimental strategy allowed us to uncover that the defective production of ATF4 and ATF4-mediated transcriptional programs is a p53-independent event that is commonly linked to ribosome biogenesis defects. Importantly, such a downregulation is found in several transformed cell lines representative of different tumor types (*TP53*^+^ and *TP53*^–^ HCT116, A549, K562), in differentiating HSPCs and in erythroid precursors derived from patients with DBA. This indicates that the downregulation of ATF4 and ATF4-regulated gene expression programs is a rather general event caused by deficits in ribosome production. We have also observed that this phenomenon takes place rapidly upon moderate losses of ribosomes (*RPS19* and *BYSL* knockdowns in HCT116 cells), indicating that the dampening of ATF4 and ATF4-dependent programs is a primary and early defect caused by partial disruptions of the ribosome synthesis machinery.

Several reasons support the idea that the downregulation of ATF4 can be an important pathobiological event driving the erythroid failure typically found in many ribosomopathies: (i) ATF4 directly activates erythroid differentiation-specific genes, including a subset of GATA1 coregulated genes,^48^ the γ-globin repressor MYB,^48^ and the terminal differentiation transcription factor GRB10^47^; (ii) the heme-regulated eIF2α kinase (HRI)–phosphorylated eIF2α–ATF4 axis is required for the expression of both redox and amino acid metabolism genes during erythropoiesis.^47^ This axis is also essential for the maintenance of erythroid cell homeostasis under low heme or iron conditions^45^^,^^47^; (iii) murine *Atf4*^−/−^ erythroid progenitors show deficits in differentiation^44^^,^^45^^,^^47^ that eventually cause fetal liver anemia^44^; (iv) our present results indicating that the loss of ATF4 caused by a ribosomal protein deficit is associated with the re-expression of γ-globin in the erythroid K562 cell line; and (v) our *in silico* analyses revealing an inverse correlation between the expression levels of ATF4-associated gene signatures and the severity of the erythroid differentiation defects found in patients with DBA. Given that ATF4 also regulates additional metabolic routes, redox pathways, the integrated stress responses, and tissue developmental decisions,^38^^,^^39^^,^^40^^,^^41^^,^^43^^,^^72^^,^^73^^,^^74^^,^^75^ it is possible that its downregulation might be associated with the developmental problems that ribosomopathy patients exhibit in other tissues outside the hematopoietic system.

Our results also indicate that ATF4 can be downregulated by two mechanisms in cells that are undergoing ribosome biogenesis defects: (i) a reduction in transcript levels, which is observed only in cells bearing defects in 40S ribosomal subunit synthesis, and (ii) a reduction in translation efficiency caused by the increase of translationally unproductive or translationally inefficient complexes, which is the primary defect in cells undergoing defects in the 60S ribosomal subunit synthesis. Against initial expectations,^62^^,^^63^ the latter defect seems to be caused by a general process that equally affects the uORFs and mORF present in the *ATF4* mRNA rather than by the preferential utilization of the uORFs by the remaining ribosomes. We surmise that both regulatory mechanisms rely on the fact that the stability of *ATF4* transcripts is dependent on the binding of 40S subunits that are either engaged in continuously scanning its rather complex 5′-UTR (composed of three uORFs), or favoring the association with stabilization factors that work similarly to LARP1 in the case of 5′-TOP mRNAs.^50^^,^^65^ According to this model, it would be expected that the stability of the *ATF4* transcript will be reduced and enhanced under conditions of low levels of free 40S subunits and high levels of free 40S subunits (which are found in cells with defects in 60S subunit production), respectively. In support of this model, we have shown that the *ATF4* mRNA is less stable and more stable in RPS19- and RPL5-deficient cells, respectively. In the same sense, it has been previously reported that the *ATF4* mRNA undergoes a decrease in stability upon mTORC1 inhibition,^43^ a treatment that inhibits ribosome subunit synthesis and, therefore, decreases the overall pool of free 40S subunits in cells. Also, in agreement with our model, the abnormal accumulation of *ATF4* transcripts not bound by ribosomes (40S-60S size-range) in RPL5-knockdown HCT116 cells is consistent with the transcripts being continuously scanned by 40S subunits that do not initiate translation due to the scarcity of 60S subunits or, alternatively, stablishing stable complexes that are translationally silenced. The differential effects of RPS (downregulation) and RPL (upregulation) deficiencies on the levels of *ATF4* mRNA can be the reason why this biological program was not detected in previous publications, given that the working hypothesis used in most of those studies was that the changes in the expression patterns of *bona-fide* ribosomopathy-associated transcripts, in terms of transcript levels and ribosome occupancies, had to be the same irrespectively of whether the disease was caused by a haploinsufficiency in RPS- or an RPL-encoding gene.

An important ramification of our study is that deficiencies in 40S ribosomal subunits will elicit a stronger effect on the output of ATF4 protein than those affecting the production of 60S ribosomal subunits. This observation raises the interesting possibility that the stronger erythroid defects found in patients with DBA bearing mutations in RPS-encoding genes^31^ could be the consequence of the more penetrant silencing of ATF4 programs. It can also provide a rational basis to explain the recent observation that patients with RPS-DBA contain a significantly higher proportion of erythroid progenitors expressing γ-globin than patients with RPL-DBA.^31^ The involvement of ATF4 in stress responses also poses the interesting possibility that the different penetrance of DBA among individuals of family members who carry identical genetic mutations can be due to different exposures to physiological stresses or infections.

### Limitations of the study

Our study clearly establishes a direct relationship between ribosome biosynthesis alterations and the downmodulation of ATF4-regulated programs. However, some limitations must be considered. One of them is that the data generated using cells from patients with DBA are based on a very low number of clinical cases. Up to now, solving this issue has been quite difficult given the low epidemiological frequency of the diseases under study. However, this problem could be solved as more patients become included in gene expression datasets. Another pending issue is to demonstrate that the re-expression of *ATF4* in erythroid cells from patients with DBA elicits the same effects found in the present study with our K562 cell model. In this sense, it will also be important to assess whether the increased expression of *ATF4* can rescue the erythroid differentiation defect of RPS19-deficient mice.^76^ Addressing these issues will require methodological improvements, since the ectopic expression of *ATF4* is not tolerated by erythroid precursors^48^ and other cell types (i.e., HCT116 cells, our unpublished observations). Finally, it will be also interesting in the future to explore whether the inducible and tissue-specific loss of ATF4 triggers a DBA-like phenotype in appropriate knock-in mouse models to provide genetic evidence for its implication in this ribosomopathy. Despite these limitations, we consider that the present results provide convincing evidence for the importance of the impairment of ATF4-dependent programs in the general cellular alterations caused by disturbances in ribosome subunit production.

## Resource availability

### Lead contact

Further information and requests for resources and reagents should be directed to and will be fulfilled by the lead contact, Mercedes Dosil (mdosil@usal.es).

### Materials availability

The availability of new generation materials associated with this article can be requested from the lead contact.

### Data and code availability

• Microarray data have been deposited at GSE256265 and are publicly available as of the date of publication. The accession number is listed in the key resources table. This paper analyzes published, publicly available RNAseq and Riboseq datasets that can be found at GSE168445, GSE89183 and GSE89540. The accession numbers for these datasets are listed also in the key resources table.

Original western blot images and the raw data of qRT-PCR analysis, growth curves and ribosome subunit quantifications have been deposited at Mendeley (https://data.mendeley.com/datasets/wvwhv7kgn6/1) and are publicly available as of the date of publication. Microscopy data reported in this paper will be shared by the lead contact upon request.

• This paper does not report original code.
• Any additional information required to reanalyze the data reported in this paper is available from the lead contact upon request.

## Acknowledgments

We thank Antonio Abad for expert technical assistance and Blanca Nieto for the Northern blot analyses. M.D. research has been financed by grants BFU2017-88192-P and PID2020-118378GB-I00, both of them co-funded by 10.13039/501100004837MCIN/10.13039/501100011033AEI/10.13039/501100011033/ plus the European Research Development Fund “A way of making Europe” of the 10.13039/501100000780European Union. X.R.B. has received funding from the Castilla-León government (CSI145P20, CSI018P23), grants co-funded by 10.13039/501100004837MCIN/10.13039/501100011033AEI/10.13039/501100011033/ plus the European Research Development Fund “A way of making Europe” of the 10.13039/501100000780European Union (PID2021-122666OB-I00, PDC2022-133027-I00, PLEC2022-009217), “la Caixa” Banking Foundation (HR20-00164), and the Programa Excelencia of the Fundación Científica AECC 2022 (EPAEC222641CICS). J.R.-V. received funding from the 10.13039/501100004587Carlos III Health Institute (PI20/01724). M.D. and X.R.B. center was supported by the Programas de Apoyo a Planes Estratégicos de Investigación de Estructuras de Investigación de Excelencia of the Castilla-León government (CLC-2017-01 and CL-EI-2021-02) that were both co-funded by the European Research Development Fund.

## Author contributions

L.F.L.-M. performed all microarray, RNA-seq, Ribo-seq gene expression data analyses, ribosome density determinations, and contributed to the design of the experiments and analysis of the results; J.R.-V. performed the ATF4 overexpression rescue experiments, the time-courses of growth, viability, ISR activation and protein expression in siRNA-transfected cells, some of the validations of gene expression changes in HCT116 cells, helped with sucrose gradient fractionation analyses and contributed to the design of the experiments; R.R.-C. carried out the microscopy analyses, PSE fractionation experiments, *p*-eIF2α analyses, preparation of RNAs for microarray analyses and some of the validations of gene expression changes in HCT116 cells. S.G.G. contributed to setting up the initial siRNA experimental conditions, performed sucrose gradient fractionation analyses, replications of gene expression validations and contributed to the analysis of the results; P.F. and A.G. contributed to the technical set-up, analysis and discussion of data from the experiments of mRNA distribution on sucrose gradients; X.R.B. contributed to the analysis of data, discussion of results and to the writing of the paper; M.D. designed the project, analyzed the data, and wrote the paper.

## Declaration of interests

The authors report no competing financial interests.

## STAR★Methods

### Key resources table

**Table:** 

REAGENT or RESOURCE	SOURCE	IDENTIFIER
**Antibodies**		

Anti-ATF4 Rabbit (D4B8)	Cell Signaling Technology	Cat# 11815; RRID: AB_2616025
HRP Anti-BRD2 antibody [EPR7642]	Abcam	Cat# ab198536
Anti-Bystin Polyclonal Antibody	Bethyl	Cat# A304-568-M; RRID: AB_2782001
Anti-GAPDH FL-335	Santa Cruz Biotechnology	Cat# 10917-2-AP; RRID:AB_10167668
Anti-GATA1 Polyclonal antibody	Proteintech	Cat# 553031; RRID: AB_2108279
Anti-p53 (1C12) Mouse mAb	Cell Signaling Technology	Cat# 2524; RRID: AB_331743
Anti-p62/SQSTM1 Antibody (2C11)	Novus Biologicals	Cat# H00008878-M01; RRID: AB_548364
Anti-Phospho-eIF2α (Ser51) Antibody	Cell Signaling Technology	Cat# 9721; RRID: AB_330951
Anti-eIF2α Antibody	BD Biosciences	Cat# 9722; RRID: AB_2230924
Anti-RPL11 antibody	Abcam	Cat# ab79352; RRID: AB_2042832
Rabbit anti-RPL5 Antibody Affinity Purified	Bethyl	Cat# A303-933-A; RRID: AB_2620282
Anti-Ribosomal Protein S19 (WW-4)	Santa Cruz Biotechnology	Cat# sc-100836; RRID: AB_1129199
Rabbit anti-RPS2 Antibody Affinity Purified	Bethyl	Cat# A303-794A; RRID: AB_11218192
Anti-RPS3 antibody	Abcam	Cat# ab140676
Rabbit anti-RPS6 Antibody	Bethyl	Cat# A300-557A; RRID: AB_477988
Anti-α-Tubulin Mouse mAb (DM1A)	Calbiochem	Cat# CP06; RRID:AB_2617116
Anti-fetal hemoglobin antibody [EPR9709]	Cell Signaling Technology	Cat# ab156584

**Bacterial and virus strains**		

DH5α competent *E. coli*	Life Technologies	Cat# 18258012

**Chemicals, peptides, and recombinant proteins**		

Lipofectamine RNAiMAX	Life Technologies	Cat# 13778150
JetPEI	Polyplus	Cat# 101-10N
Lenti-X™ concentrator	Takara	Cat# 631231
DAPI	Thermo Fisher Scientific	Cat# D1306
TO-PRO-3	Thermo Fisher Scientific	Cat# T3605
Polybrene	Sigma-Aldrich	Cat# H9268-5G
Doxycycline	Sigma-Aldrich	Cat# D9891-1G
Puromycin	Sigma	Cat# P9620
cØmplete, protease inhibitor cocktail	Roche	Cat# 05056489001
NZYol	NZYtech	Cat# MB18502
Cycloheximide	Sigma-Aldrich	Cat# C7698
RNasin, ribonuclease inhibitor	Promega	Cat# N2111
alpha-amanitin	Sigma-Aldrich	Cat# A2263-1MG

**Critical commercial assays**		

Neon™ Transfection System 100 μL Kit	ThermoFisher Scientific	Cat# MPK10096
RNAeasy Mini Kit	Qiagen	Cat# 74104
iScript One-Step RT-PCR kit with Syber Green	BioRad	Cat# 4389986
iQ SYBR Green Supermix	BioRad	Cat# 1708882
CFBlue Annexin V Apoptosis Detection Kit with 7-AAD	Immunostep	Cat# ANXVKCFB7-100T

**Deposited data**		

Microarray data	This study	GEO: GSE256265
RNA-seq data	Luan et al.^66^	GEO: GSE168445
RNA-seq data	Khajuria et al.^32^	GEO: GSE89183
Microarray data	O'Brien et al.^33^	GEO: GSE89540
Raw data	This study	https://data.mendeley.com/datasets/wvwhv7kgn6/1

**Experimental models: Cell lines**		

HCT116 TP53+/+	Dra. M. Sacristán Lab	N/A
HCT116 TP53–/–	Dra. M. Sacristán Lab	N/A
HeLa	ATCC	Cat# CCL-2, RRID: CVCL_0030
K562	Dr. C. Guerrero Lab	N/A

**Oligonucleotides**		

Oligonucleotides: see Table S1	This study	N/A
siRNAs: see Table S2	This study	N/A

**Recombinant DNA**		

pFG42 TRE-FLAG-ATF4 UbC-rtTA-IRES-GFP	Dr. D. Raulet Lab	N/A

**Software and algorithms**		

FlowJo (version 10.8.2)	FlowJo, LLC	https://www.flowjo.com/solutions/flowjo
R (version 3.6.3)	R Core Team	https://www.R-project.org/
Limma	Richie et al.^77^	https://bioconductor.org/packages/release/bioc/html/limma.html
DAVID	Sherman et al.^78^	https://david.ncifcrf.gov
Heatmap3	Zhao et al.^79^	http://CRAN.R-project.org/package=heatmap3
GSEA	Subramanian et al.^80^	http://software.broadinstitute.org/gsea/index.jsp
ssGSEA	Subramanian et al.,^80^ Reich et al.^81^	https://genepattern.broadinstitute.org/gp/pages/login.jsf
STAR (version 2.7.6a)	Dobin et al.^82^	https://bioweb.pasteur.fr/packages/pack@STAR@2.7.6a
Samtools (version 1.14)	Danecek et al.^83^	http://www.htslib.org
StepOne software (version 2.1)	ThermoFisher Scientific	https://www.thermofisher.com/order/catalog/product/4376600
ImageJ (version 1.44p)	NIH Image	https://imagej.nih.gov/ij/
GraphPad Prism (version 6.0)	GraphPad Software Inc	https://www.graphpad.com/scientific-software/prism/

### Experimental model and study participant details

#### Cell lines

The HCT116 TP53+/+ and HCT116 TP53–/– lines were kindly provided by professor María Sacristán of Centro de Investigación del Cáncer of Salamanca. The HeLa cell line was obtained from ATCC. These cell lines were cultured in Dulbecco’s modified Eagle’s medium (DMEM) supplemented with 10% fetal bovine serum, 100 U/ml penicillin/streptomycin, 2 mM L-Glutamine and maintained under standard tissue culture conditions. The K562 cell line was kindly provided by Professor Carmen Guerrero of Centro de Investigación del Cáncer of Salamanca. K562 cells were cultured in RPMI1640 medium supplemented with 10% fetal bovine serum, 100 U/ml penicillin/streptomycin and maintained at 37°C in incubators with 5% CO_2_ atmosphere. All culture reagents were obtained from Gibco-Thermo Fisher Scientific. All cell lines were tested biweekly for mycoplasma contamination (Cat. No. hb-det2, Invivogen). The cell lines have not been authenticated since their original receipt. All cell lines were tested biweekly for mycoplasma contamination (Cat. No. hb-det2, Invivogen), confirming negative results. Sample size calculation was not performed in this study.

#### Human samples

This article uses publicly available gene expression profiling data from human samples. Access numbers to the datasets are listed in the key resources table and in the data availability and codes section. We have not directly manipulated any human samples.

### Method details

#### siRNA-mediated transcript knockdowns

For siRNA mediated knockdowns, HCT116 cells were reverse transfected with 10 nM of the siRNA duplexes (listed in Table S2) using 8 μL/mL of Lipofectamine RNAiMAX (Cat No. 13778150, Life Technologies) as previously described.^49^ For the knockdowns in the K562 line, cells were transfected with 20 nM of the appropriate siRNA duplexes (diluted in 100 μL of R buffer; Cat No. MPK10096, Life Technologies) using three 10-msec electroporation cycles at 1.45 mV in the Neon system (Life Technologies). The siRNA duplexes were purchased from Ambion-Thermo Fisher Scientific (Silencer Select siRNA). Cells were harvested 24, 48 or 72 hours after transfection, as indicated in the figures. Negative controls were either untreated cells or cells transfected with a control scrambled (SCR) siRNA (Cat. No. 4390844, Ambion-Thermo Fisher Scientific).

#### Production of lentiviral particles

To generate infectious lentiviral particles, we transfected the lentiviral plasmids together with the p-vsv-g and p-pax2 packaging vectors into HEK293T cells using the JetPEI transfection reagent (Cat. No. 101-10N, Polyplus). Lentivirus-containing supernatants were collected and concentrated using Lenti-X™ concentrator (Cat. No. 631231, Takara) by centrifugation at 2,000 xg for 1 hour at 4°C. Concentrated viruses were resuspended and tittered using infection of NIH3T3 cells and scoring GFP-positive cells by flow cytometry. Viruses with high titers were aliquoted and stored in –80°C for up to 2 months.

#### Preparation of whole-cell and fractionated lysates

For whole-cell lysates, cells were washed three times with phosphate-buffered saline solution and lysed in RIPA buffer (10 mM Tris-HCl [pH 8.0], 150 mM NaCl, 1% Triton X100, 5 mM NaF, 1 mM Na3VO4, 1 mM β-glycerol phosphate, supplemented with a cocktail of protease inhibitors [Cømplete, Cat. No. 05056489001, Roche]). The lysates were precleared by centrifugation at 20,000 xg for 10 min at 4°C, and protein concentration was determined with Precision Red reagent (Cat. No. ADV02-A, Cytoskeleton) following the manufacturer directions. For the preparation of the SN1, SN2 and SN3 lysate fractions obtained with the Preribosome Sequential Extraction (PSE) method, we followed a previously published detailed protocol^58^ with one additional step to get a sample of the insoluble material that is ten-fold concentrated. In brief, the pellet remaining after the SN3 extraction step was resuspended in 80 μl of SDS-PAGE loading buffer and a 24 μl aliquot [labeled as sample P(10x)] was taken to analyze by western blot together with the SN1, SN2 and SN3 samples.

#### Western blot analyses

Protein extracts were separated electrophoretically and transferred onto nitrocellulose filters (Thermo Fisher Scientific) using the iBlot Dry Blotting System (Thermo Fisher Scientific). Membranes were blocked in 5% dry milk in TBS-T (25 mM Tris-HCl [pH 8.0], 150 mM NaCl, 0.1% Tween-20) for at least 1 hour and then incubated overnight with the appropriate antibodies. Membranes were then washed three times with TBS-T, incubated with the appropriate secondary antibody for 30 min at room temperature, and washed twice as above. Immunoreacting bands were detected by horseradish peroxidase- conjugated secondary antibodies to rabbit (Cat. No. GENA934-1ML, Cytiva-Merck) and mouse (Cat. No. GENA931-1ML, Cytiva-Merck) immunoglobulins and the Pierce ECL Western Blotting Substrate (Cat. No. #RPN2106, Amersham). The sources and dilutions of primary antibodies are shown in key resources table.

#### Indirect immunofluorescence and confocal microscopy

For the analysis of BRD2 subcellular localization by immunofluorescence, HeLa cells were fixed in PBS containing 4% paraformaldehyde (Cat. No. 1040021000, Merck) and permeabilized for 10 min in 0.5 % Triton X-100 (Cat. No. X-100, Sigma-Merck) in TBS- T (20mM Tris-HCl [pH 7.5], 150mM NaCl, 0.1% Tween-20). After blocking with 2% bovine serum albumin (Cat. No. A2153, Sigma-Merck) for 30 min, coverslips were incubated with the primary antibody for 2 h at room temperature. Preparations were washed four times with TBS-T, incubated with the secondary antibody for 45 min, and stained with DAPI (4′,6-diamidino-2-phenylindole) (Cat. No. D1306, Thermo Fisher Scientific) before being mounted in Mowiol. Information about the BRD2 antibody is shown in key resources table. The analysis of NOC4L nucleolar localization were performed on a previously generated cell line that endogenously expresses the NOC4L-GFP fusion protein from the endogenous *NOC4L* locus.^56^ Cells were fixed at the appropriate time points after si-RNA transfections, stained with TO-PRO-3 (Cat. No. T3605, Thermo Fisher Scientific), and mounted onto Mowiol prior to microscopy observation. Imaging was performed on Leica TCS SP8 X (Leica Microsystems) confocal microscope, driven by the LAS-XTM version 3.1.5 16308 software, using a x63/1.4 oil immersion optical lens (HC PL APO SC2) (optical section: 0.896 μm). GFP and TO- PRO-3 samples were excited with a pulsed white light laser at 488 and 641 nm, respectively. GFP images were acquired using a Leica HyD reflected light detector and TO-PRO-3 images with a photomultiplier tube.

#### Northern blot analyses

Preparation of total cellular RNAs and northern blotting were performed following detailed procedures described previously.^58^ The sequence of the 5'-ITS1 probe is shown in Table S3.

#### Determination of cell proliferation

HCT116 TP53+/+, HCT116 TP53–/– and K562 cells were transfected with the indicated siRNA and proliferation was measured in all cases using the 3-(4,5-dimethylthiazol-2-yl) 2,5-diphenyltetrazolium bromide (MTT) method at the indicated time points. To this end, the culture medium of each well was replaced by 100 μl of the MTT solution (0.5 mg/ml) made in phosphate-buffered saline solution. After 2 hr at 37°C in a 5% CO_2_ atmosphere, 100 μl of DMSO were added per well to dissolve the formazan crystals formed and the absorbance at 570 nm measured 15 min later using the Ultraevolution reader.

#### Determination of apoptotic rates

HCT116 TP53+/+, HCT116 TP53–/– and K562 cells were harvested 48 h after the siRNA transfection, stained using the CFBlue Annexin V Apoptosis Detection Kit with 7-AAD (Cat. No. ANXVKCFB7-100T, Immunostep), and apoptosis determined using flow cytometry.

#### Analysis of polysome profiles and mRNA distribution

The distribution of mRNAs in the different polysome complexes was analyzed following the protocol described by Fuentes et al.^65^ Briefly, 1·10^6^ HCT116 cells were reverse transfected with the appropriate siRNA in 100-mm dishes. After 24 hours, cells were incubated with 0.1 mg/ml cycloheximide (CHX) in the tissue culture incubator for 5 min. The cells were then washed twice with cold PBS containing 0.1 mg/ml cycloheximide, scraped on ice, and centrifuged at 200 xg for 5 min. Polysome lysates were prepared by resuspension of cell pellets in 250 μl of hypotonic lysis buffer [1.5 mM KCl, 2.5 mM MgCl_2_, 5 mM Tris-HCl (pH 7.4), 1 mM dithiothreitol (DTT), 1 % sodium deoxycholate, 1 % Triton X-100, and CHX (0.1 mg/ml), supplemented with a cocktail of protease inhibitors (cØmplete, Cat. No. 05056489001, Roche) and RNase inhibitor (RNasin, Cat. No. N2111, Promega)], and incubated on ice for 5 min. Cell lysates were cleared by centrifugation at 20,000 xg for 5 min. Protein concentrations were determined using Precision Red reagent (Cat. No. ADV01, Cytoskeleton), and 0.8 mg of polysomal lysates were loaded onto linear 10% to 50% sucrose gradients containing 80 mM NaCl, 5 mM MgCl_2_, 20 mM Tris-HCl (pH 7.4), 1 mM DTT, and RNase inhibitor (10 U/ml). Gradient ultracentrifugation was performed on a SW40-Ti (Beckman) rotor at 218000 xg for 2 hours and 55 min at 4°C. Gradients were analysed on a Brandel BR- 186 gradient fractionator with syringe pump coupled to a Spectra/Chrom 280 UV monitor and chart recorder and collected in 13 fractions ranging from light to heavy sucrose. SDS was added to each fraction to a final concentration of 1% and samples were incubated at 65°C for 10 min. Prior to total RNA extraction, 1 ng of firefly luciferase mRNA (Cat. No. L456A, Promega) was added to each fraction, followed by phenol-chloroform extraction and precipitation with isopropanol. cDNAs were obtained from purified the RNA contained in each fraction using random primers and SuperScript II reverse transcriptase (Cat. No. 18064014, ThermoFisher) according to the manufacturer’s guidelines. mRNAs were quantified employing iQ SYBR Green Supermix (Cat. No. 1708882, BioRad) and normalized to firefly mRNA. The specific primers used in qPCR experiments are listed in Table S3.

#### Transcriptome and translatome analyses

Two independent experiments were performed to analyze the eight conditions subject to microarray analyses: HCT116 *TP53*^*+/+*^ and HCT116 *TP53*^*–/–*^ cells harvested 24 hours (si- ctrl and si-RPS19) and 48 hours (si-ctrl and si-BYSL) after siRNA transfection. Each experiment included three biological replicates of each condition. Total RNAs were isolated using the RNAeasy Mini Kit (Cat. No. 74104, Qiagen) and analyzed using the Affymetrix platform (Clariom™ S Assay HT) at the CIC Genomics Core Facility according to the manufacture's recommendations. R (version 3.6.3) was used to perform the bioinformatic analyses. Signal intensity values were obtained from CEL files after applying the Robust Multichip Average function for background adjustment, quantile normalization and summarization.^84^ Differentially expressed genes were identified using linear models for microarray data (limma)^77^ and adjusting *P* values for multiple comparisons by applying the Benjamini-Hochberg correction method (FDR).^85^ A FDR q-value of 0.05 and a fold-change of 50% were set as thresholds for statistical significance. The heatmap3 package^79^ was used to generate the heatmaps. Gene Ontology and KEGG pathways enrichment analyses were performed using DAVID.^78^ Gene Set Enrichment Analysis (GSEA)^80^ was carried out with the described gene sets using gene set permutations (n = 1,000) for the assessment of significance and signal-to-noise metric for ranking genes.^74^ Single-sample Gene Set Enrichment Analysis (ssGSEA)^80^^,^^81^ was used to calculate the score of gene signatures across individual samples. The gene sets used for (ss)GSEA analyses were obtained from the Molecular Signatures Database (MSigDB, v7.4),^86^ with the exception of the AmTOR signature that includes 41 genes induced by mTORC1 in an ATF4-dependent manner that were selected from a previously published study.^40^ These gene sets were evaluated in the samples generated in this work and/or publicly available datasets accessed through Gene Expression Omnibus (GEO): GSE168445,^66^ GSE89183,^32^ and GSE89540.^33^ ATF4 direct targets were established based on the data available at ChIP-Atlas^68^ (erythroblasts, distance to TSS < 5 kb). Ribosome occupancy on *ATF4* mRNA (NM_182810.3) was calculated on the indicated samples from the dataset GSE168445^66^ using STAR^82^ (version 2.7.6a) for read alignment to the human genome (GRCh38) and samtools^83^ (version 1.14) for depth calculation.

#### ATF4 overexpression rescue experiments

For doxycycline-inducible ATF4 expression in K562, the construct used was pFG42 TRE-FLAG-ATF4 UbC-rtTA-IRES-GFP (a kind gift from David Raulet at the University of California, Berkeley).^87^ K562 cells were transduced in the presence of polybrene (6 μg/mL; Cat. No. H9268-5G, Sigma-Aldrich) with lentiviral supernatants and pelleted by centrifugation (2,000 xg for 60 min). The transduction efficiency was examined by flow cytometry 48 hours after transduction and GFP-positive cells were sorted. Transduced cells were then cultured with 100 ng/ml of doxycycline (Cat. No. D9891-1G, Sigma- Aldrich D9891-1G) and 24 hours later transfected with the appropriate siRNA duplexes and maintained in medium with doxycycline for 72 hours before being harvested for analysis.

#### Quantitative RT-PCR

Total RNA was extracted from cells using NZYol (Cat. No. MB18502, NZYtech) and mRNAs purified using RNeasy Mini kit (Cat. No. 74104, Qiagen) following the manufacturer's guidelines. mRNA abundance was analysed by qRT-PCR using the iScript One-Step RT-PCR kit with SYBR green (Cat. No. 4389986, Applied BioSystems) and the StepOnePlus Real-Time PCR System (Cat. No. 4376600, Applied BioSystems). Raw qRT-PCR data were analysed using the StepOne software v2.1 (Applied Biosystems), using the abundance of the endogenous *Gapdh* as internal normalization control. Primers used for transcript quantitation are listed in Table S3.

#### RNA half-life measurement

HCT116 cells were transfected with 10 nM of the siRNA duplexes as previously described and plated in a six-well plate. Cells were treated with 5 μg/mL of alpha-amanitin (Cat. No. A2263-1MG, Sigma) and total RNA was extracted at time-points 0, 4, 8, 14, and 24 hours post-incubation. RNA levels were analyzed by RT-qPCR as described above using gene-specific oligonucleotides indicated in Table S3.

### Quantifications and statistical analysis

Calculations were performed using Microsoft Excel 2020 and GraphPad Prism software (version 6.0). The number of biological replicates (n), the type of statistical tests performed, and the statistical significance are indicated for each experiment in the figure legends and/or the results section of this document. Data normality and equality of variances were analyzed with Shapiro-Wilk and Bartlett’s tests, respectively. Parametric distributions were analyzed using Student’s t-test (when comparing two experimental groups) or ANOVA followed by either Dunnett’s (when comparing more than two experimental groups with a single control group). Nonparametric distributions were analyzed using the Kruskal-Wallis followed by Dunn’s tests. In all cases, values were considered significant when *P* ≤ 0.05 and data are given as the mean ± SEM.

## Supplemental information

Document S1. Figures S1–S5 and Tables S2 and S3

Table S1. Excel table containing the lists of differentially expressed genes in RPS19- and Bystin-depleted HCT116 cells

## References

[1] Baßler J., Hurt E. (2019). Eukaryotic Ribosome Assembly. Annu. Rev. Biochem..

[2] Kressler D., Hurt E., Baßler J. (2017). A Puzzle of Life: Crafting Ribosomal Subunits. Trends Biochem. Sci..

[3] Klinge S., Woolford J.L. (2019). Ribosome assembly coming into focus. Nat. Rev. Mol. Cell Biol..

[4] Dorner K., Ruggeri C., Zemp I., Kutay U. (2023). Ribosome biogenesis factors-from names to functions. EMBO J..

[5] Ni C., Buszczak M. (2023). The homeostatic regulation of ribosome biogenesis. Semin. Cell Dev. Biol..

[6] Bustelo X.R., Dosil M. (2018). Ribosome biogenesis and cancer: basic and translational challenges. Curr. Opin. Genet. Dev..

[7] de la Cruz J., Karbstein K., Woolford J.L. (2015). Functions of ribosomal proteins in assembly of eukaryotic ribosomes *in vivo*. Annu. Rev. Biochem..

[8] Danilova N., Gazda H.T. (2015). Ribosomopathies: how a common root can cause a tree of pathologies. Dis. Model. Mech..

[9] Farley-Barnes K.I., Ogawa L.M., Baserga S.J. (2019). Ribosomopathies: Old Concepts, New Controversies. Trends Genet..

[10] Kampen K.R., Sulima S.O., Vereecke S., De Keersmaecker K. (2020). Hallmarks of ribosomopathies. Nucleic Acids Res..

[11] Pelletier J., Thomas G., Volarević S. (2018). Ribosome biogenesis in cancer: new players and therapeutic avenues. Nat. Rev. Cancer.

[12] Aspesi A., Ellis S.R. (2019). Rare ribosomopathies: insights into mechanisms of cancer. Nat. Rev. Cancer.

[13] Mills E.W., Green R. (2017). Ribosomopathies: There's strength in numbers. Science.

[14] Kang J., Brajanovski N., Chan K.T., Xuan J., Pearson R.B., Sanij E. (2021). Ribosomal proteins and human diseases: molecular mechanisms and targeted therapy. Signal Transduct. Target. Ther..

[15] Fuentes P., Pelletier J., Gentilella A. (2024). Decoding ribosome complexity: role of ribosomal proteins in cancer and disease. NAR Cancer.

[16] Gazda H.T., Sheen M.R., Vlachos A., Choesmel V., O'Donohue M.F., Schneider H., Darras N., Hasman C., Sieff C.A., Newburger P.E. (2008). Ribosomal protein L5 and L11 mutations are associated with cleft palate and abnormal thumbs in Diamond-Blackfan anemia patients. Am. J. Hum. Genet..

[17] Vlachos A., Blanc L., Lipton J.M. (2014). Diamond Blackfan anemia: a model for the translational approach to understanding human disease. Expert Rev. Hematol..

[18] Vlachos A., Rosenberg P.S., Atsidaftos E., Alter B.P., Lipton J.M. (2012). Incidence of neoplasia in Diamond Blackfan anemia: a report from the Diamond Blackfan Anemia Registry. Blood.

[19] Alter B.P., Giri N., Savage S.A., Rosenberg P.S. (2018). Cancer in the National Cancer Institute inherited bone marrow failure syndrome cohort after fifteen years of follow-up. Haematologica.

[20] Vlachos A., Rosenberg P.S., Atsidaftos E., Kang J., Onel K., Sharaf R.N., Alter B.P., Lipton J.M. (2018). Increased risk of colon cancer and osteogenic sarcoma in Diamond-Blackfan anemia. Blood.

[21] Da Costa L., Leblanc T., Mohandas N. (2020). Diamond-Blackfan anemia. Blood.

[22] Ulirsch J.C., Verboon J.M., Kazerounian S., Guo M.H., Yuan D., Ludwig L.S., Handsaker R.E., Abdulhay N.J., Fiorini C., Genovese G. (2019). The Genetic Landscape of Diamond-Blackfan Anemia. Am. J. Hum. Genet..

[23] Golomb L., Volarevic S., Oren M. (2014). p53 and ribosome biogenesis stress: the essentials. FEBS Lett..

[24] Liu Y.L., Shibuya A., Glader B., Wilkes M.C., Barna M., Sakamoto K.M. (2023). Animal models of Diamond-Blackfan anemia: updates and challenges. Haematologica.

[25] Torihara H., Uechi T., Chakraborty A., Shinya M., Sakai N., Kenmochi N. (2011). Erythropoiesis failure due to RPS19 deficiency is independent of an activated Tp53 response in a zebrafish model of Diamond-Blackfan anaemia. Br. J. Haematol..

[26] Yadav G.V., Chakraborty A., Uechi T., Kenmochi N. (2014). Ribosomal protein deficiency causes Tp53-independent erythropoiesis failure in zebrafish. Int. J. Biochem. Cell Biol..

[27] Singh S.A., Goldberg T.A., Henson A.L., Husain-Krautter S., Nihrane A., Blanc L., Ellis S.R., Lipton J.M., Liu J.M. (2014). p53-Independent cell cycle and erythroid differentiation defects in murine embryonic stem cells haploinsufficient for Diamond Blackfan anemia-proteins: RPS19 versus RPL5. PLoS One.

[28] Morgado-Palacin L., Varetti G., Llanos S., Gómez-López G., Martinez D., Serrano M. (2015). Partial Loss of Rpl11 in Adult Mice Recapitulates Diamond-Blackfan Anemia and Promotes Lymphomagenesis. Cell Rep..

[29] Ludwig L.S., Gazda H.T., Eng J.C., Eichhorn S.W., Thiru P., Ghazvinian R., George T.I., Gotlib J.R., Beggs A.H., Sieff C.A. (2014). Altered translation of GATA1 in Diamond-Blackfan anemia. Nat. Med..

[30] Boussaid I., Le Goff S., Floquet C., Gautier E.F., Raimbault A., Viailly P.J., Al Dulaimi D., Burroni B., Dusanter-Fourt I., Hatin I. (2021). Integrated analyses of translatome and proteome identify the rules of translation selectivity in RPS14-deficient cells. Haematologica.

[31] Iskander D., Wang G., Heuston E.F., Christodoulidou C., Psaila B., Ponnusamy K., Ren H., Mokhtari Z., Robinson M., Chaidos A. (2021). Single-cell profiling of human bone marrow progenitors reveals mechanisms of failing erythropoiesis in Diamond-Blackfan anemia. Sci. Transl. Med..

[32] Khajuria R.K., Munschauer M., Ulirsch J.C., Fiorini C., Ludwig L.S., McFarland S.K., Abdulhay N.J., Specht H., Keshishian H., Mani D.R. (2018). Ribosome Levels Selectively Regulate Translation and Lineage Commitment in Human Hematopoiesis. Cell.

[33] O'Brien K.A., Farrar J.E., Vlachos A., Anderson S.M., Tsujiura C.A., Lichtenberg J., Blanc L., Atsidaftos E., Elkahloun A., An X. (2017). Molecular convergence in ex vivo models of Diamond-Blackfan anemia. Blood.

[34] Horos R., Ijspeert H., Pospisilova D., Sendtner R., Andrieu-Soler C., Taskesen E., Nieradka A., Cmejla R., Sendtner M., Touw I.P., von Lindern M. (2012). Ribosomal deficiencies in Diamond-Blackfan anemia impair translation of transcripts essential for differentiation of murine and human erythroblasts. Blood.

[35] Bibikova E., Youn M.Y., Danilova N., Ono-Uruga Y., Konto-Ghiorghi Y., Ochoa R., Narla A., Glader B., Lin S., Sakamoto K.M. (2014). TNF-mediated inflammation represses GATA1 and activates p38 MAP kinase in RPS19-deficient hematopoietic progenitors. Blood.

[36] Kapralova K., Jahoda O., Koralkova P., Gursky J., Lanikova L., Pospisilova D., Divoky V., Horvathova M. (2020). Oxidative DNA Damage, Inflammatory Signature, and Altered Erythrocytes Properties in Diamond-Blackfan Anemia. Int. J. Mol. Sci..

[37] Quarello P., Garelli E., Carando A., Cillario R., Brusco A., Giorgio E., Ferrante D., Corti P., Zecca M., Luciani M. (2020). A 20-year long term experience of the Italian Diamond-Blackfan Anaemia Registry: RPS and RPL genes, different faces of the same disease?. Br. J. Haematol..

[38] Pakos-Zebrucka K., Koryga I., Mnich K., Ljujic M., Samali A., Gorman A.M. (2016). The integrated stress response. EMBO Rep..

[39] Walter P., Ron D. (2011). The unfolded protein response: from stress pathway to homeostatic regulation. Science.

[40] Torrence M.E., MacArthur M.R., Hosios A.M., Valvezan A.J., Asara J.M., Mitchell J.R., Manning B.D. (2021). The mTORC1-mediated activation of ATF4 promotes protein and glutathione synthesis downstream of growth signals. Elife.

[41] Ben-Sahra I., Hoxhaj G., Ricoult S.J.H., Asara J.M., Manning B.D. (2016). mTORC1 induces purine synthesis through control of the mitochondrial tetrahydrofolate cycle. Science.

[42] Adams C.M. (2007). Role of the transcription factor ATF4 in the anabolic actions of insulin and the anti-anabolic actions of glucocorticoids. J. Biol. Chem..

[43] Park Y., Reyna-Neyra A., Philippe L., Thoreen C.C. (2017). mTORC1 Balances Cellular Amino Acid Supply with Demand for Protein Synthesis through Post-transcriptional Control of ATF4. Cell Rep..

[44] Masuoka H.C., Townes T.M. (2002). Targeted disruption of the activating transcription factor 4 gene results in severe fetal anemia in mice. Blood.

[45] Suragani R.N.V.S., Zachariah R.S., Velazquez J.G., Liu S., Sun C.W., Townes T.M., Chen J.J. (2012). Heme-regulated eIF2alpha kinase activated Atf4 signaling pathway in oxidative stress and erythropoiesis. Blood.

[46] Chen J.J., Zhang S. (2019). Heme-regulated eIF2alpha kinase in erythropoiesis and hemoglobinopathies. Blood.

[47] Zhang S., Macias-Garcia A., Ulirsch J.C., Velazquez J., Butty V.L., Levine S.S., Sankaran V.G., Chen J.J. (2019). HRI coordinates translation necessary for protein homeostasis and mitochondrial function in erythropoiesis. Elife.

[48] Boontanrart M.Y., Schröder M.S., Stehli G.M., Banović M., Wyman S.K., Lew R.J., Bordi M., Gowen B.G., DeWitt M.A., Corn J.E. (2020). ATF4 Regulates MYB to Increase gamma-Globin in Response to Loss of beta-Globin. Cell Rep..

[49] Tafforeau L., Zorbas C., Langhendries J.L., Mullineux S.T., Stamatopoulou V., Mullier R., Wacheul L., Lafontaine D.L.J. (2013). The complexity of human ribosome biogenesis revealed by systematic nucleolar screening of Pre-rRNA processing factors. Mol. Cell.

[50] Gentilella A., Moron-Duran F.D., Fuentes P., Zweig-Rocha G., Riano-Canalias F., Pelletier J., Ruiz M., Turon G., Castano J., Tauler A. (2017). Autogenous Control of 5'TOP mRNA Stability by 40S Ribosomes. Mol. Cell.

[51] Sur S., Pagliarini R., Bunz F., Rago C., Diaz L.A., Kinzler K.W., Vogelstein B., Papadopoulos N. (2009). A panel of isogenic human cancer cells suggests a therapeutic approach for cancers with inactivated p53. Proc. Natl. Acad. Sci. USA.

[52] Choesmel V., Bacqueville D., Rouquette J., Noaillac-Depeyre J., Fribourg S., Crétien A., Leblanc T., Tchernia G., Da Costa L., Gleizes P.E. (2007). Impaired ribosome biogenesis in Diamond-Blackfan anemia. Blood.

[53] O'Donohue M.F., Choesmel V., Faubladier M., Fichant G., Gleizes P.E. (2010). Functional dichotomy of ribosomal proteins during the synthesis of mammalian 40S ribosomal subunits. J. Cell Biol..

[54] Nicolas E., Parisot P., Pinto-Monteiro C., de Walque R., De Vleeschouwer C., Lafontaine D.L.J. (2016). Involvement of human ribosomal proteins in nucleolar structure and p53-dependent nucleolar stress. Nat. Commun..

[55] Carron C., O'Donohue M.F., Choesmel V., Faubladier M., Gleizes P.E. (2011). Analysis of two human pre-ribosomal factors, bystin and hTsr1, highlights differences in evolution of ribosome biogenesis between yeast and mammals. Nucleic Acids Res..

[56] Nieto B., Gaspar S.G., Moriggi G., Pestov D.G., Bustelo X.R., Dosil M. (2020). Identification of distinct maturation steps involved in human 40S ribosomal subunit biosynthesis. Nat. Commun..

[57] Frottin F., Schueder F., Tiwary S., Gupta R., Körner R., Schlichthaerle T., Cox J., Jungmann R., Hartl F.U., Hipp M.S. (2019). The nucleolus functions as a phase-separated protein quality control compartment. Science.

[58] Nieto B., Gaspar S.G., Sapio R.T., Clavaín L., Bustelo X.R., Pestov D.G., Dosil M. (2021). Efficient fractionation and analysis of ribosome assembly intermediates in human cells. RNA Biol..

[59] Maddocks O.D.K., Vousden K.H. (2011). Metabolic regulation by p53. J. Mol. Med..

[60] Pesciotta E.N., Lam H.S., Kossenkov A., Ge J., Showe L.C., Mason P.J., Bessler M., Speicher D.W. (2015). In-Depth, Label-Free Analysis of the Erythrocyte Cytoplasmic Proteome in Diamond Blackfan Anemia Identifies a Unique Inflammatory Signature. PLoS One.

[61] Danilova N., Wilkes M., Bibikova E., Youn M.Y., Sakamoto K.M., Lin S. (2018). Innate immune system activation in zebrafish and cellular models of Diamond Blackfan Anemia. Sci. Rep..

[62] Lu P.D., Harding H.P., Ron D. (2004). Translation reinitiation at alternative open reading frames regulates gene expression in an integrated stress response. J. Cell Biol..

[63] Vattem K.M., Wek R.C. (2004). Reinitiation involving upstream ORFs regulates ATF4 mRNA translation in mammalian cells. Proc. Natl. Acad. Sci. USA.

[64] Harding H.P., Novoa I., Zhang Y., Zeng H., Wek R., Schapira M., Ron D. (2000). Regulated translation initiation controls stress-induced gene expression in mammalian cells. Mol. Cell.

[65] Fuentes P., Pelletier J., Martinez-Herráez C., Diez-Obrero V., Iannizzotto F., Rubio T., Garcia-Cajide M., Menoyo S., Moreno V., Salazar R. (2021). The 40S-LARP1 complex reprograms the cellular translatome upon mTOR inhibition to preserve the protein synthetic capacity. Sci. Adv..

[66] Luan Y., Tang N., Yang J., Liu S., Cheng C., Wang Y., Chen C., Guo Y.N., Wang H., Zhao W. (2022). Deficiency of ribosomal proteins reshapes the transcriptional and translational landscape in human cells. Nucleic Acids Res..

[67] Andreev D.E., O'Connor P.B.F., Fahey C., Kenny E.M., Terenin I.M., Dmitriev S.E., Cormican P., Morris D.W., Shatsky I.N., Baranov P.V. (2015). Translation of 5' leaders is pervasive in genes resistant to eIF2 repression. Elife.

[68] Zou Z., Ohta T., Miura F., Oki S. (2022). ChIP-Atlas 2021 update: a data-mining suite for exploring epigenomic landscapes by fully integrating ChIP-seq, ATAC-seq and Bisulfite-seq data. Nucleic Acids Res..

[69] Huang P., Peslak S.A., Lan X., Khandros E., Yano J.A., Sharma M., Keller C.A., Giardine B., Qin K., Abdulmalik O. (2020). The HRI-regulated transcription factor ATF4 activates BCL11A transcription to silence fetal hemoglobin expression. Blood.

[70] Rio S., Gastou M., Karboul N., Derman R., Suriyun T., Manceau H., Leblanc T., El Benna J., Schmitt C., Azouzi S. (2019). Regulation of globin-heme balance in Diamond-Blackfan anemia by HSP70/GATA1. Blood.

[71] Yang Z., Keel S.B., Shimamura A., Liu L., Gerds A.T., Li H.Y., Wood B.L., Scott B.L., Abkowitz J.L. (2016). Delayed globin synthesis leads to excess heme and the macrocytic anemia of Diamond Blackfan anemia and del(5q) myelodysplastic syndrome. Sci. Transl. Med..

[72] Zhao Y., Zhou J., Liu D., Dong F., Cheng H., Wang W., Pang Y., Wang Y., Mu X., Ni Y. (2015). ATF4 plays a pivotal role in the development of functional hematopoietic stem cells in mouse fetal liver. Blood.

[73] Liu J., Pasini S., Shelanski M.L., Greene L.A. (2014). Activating transcription factor 4 (ATF4) modulates post-synaptic development and dendritic spine morphology. Front. Cell. Neurosci..

[74] Wang W., Lian N., Li L., Moss H.E., Wang W., Perrien D.S., Elefteriou F., Yang X. (2009). Atf4 regulates chondrocyte proliferation and differentiation during endochondral ossification by activating Ihh transcription. Development.

[75] Ritter B., Zschüntsch J., Kvachnina E., Zhang W., Ponimaskin E.G. (2004). The GABA(B) receptor subunits R1 and R2 interact differentially with the activation transcription factor ATF4 in mouse brain during the postnatal development. Brain Res. Dev. Brain Res..

[76] Jaako P., Flygare J., Olsson K., Quere R., Ehinger M., Henson A., Ellis S., Schambach A., Baum C., Richter J. (2011). Mice with ribosomal protein S19 deficiency develop bone marrow failure and symptoms like patients with Diamond-Blackfan anemia. Blood.

[77] Ritchie M.E., Phipson B., Wu D., Hu Y., Law C.W., Shi W., Smyth G.K. (2015). limma powers differential expression analyses for RNA-sequencing and microarray studies. Nucleic Acids Res..

[78] Sherman B.T., Hao M., Qiu J., Jiao X., Baseler M.W., Lane H.C., Imamichi T., Chang W. (2022). DAVID: a web server for functional enrichment analysis and functional annotation of gene lists (2021 update). Nucleic Acids Res..

[79] Zhao S., Guo Y., Sheng Q., Shyr Y. (2014). Advanced heat map and clustering analysis using heatmap3. BioMed Res. Int..

[80] Subramanian A., Tamayo P., Mootha V.K., Mukherjee S., Ebert B.L., Gillette M.A., Paulovich A., Pomeroy S.L., Golub T.R., Lander E.S., Mesirov J.P. (2005). Gene set enrichment analysis: a knowledge-based approach for interpreting genome-wide expression profiles. Proc. Natl. Acad. Sci. USA.

[81] Reich M., Liefeld T., Gould J., Lerner J., Tamayo P., Mesirov J.P. (2006). GenePattern 2.0. Nat. Genet..

[82] Dobin A., Davis C.A., Schlesinger F., Drenkow J., Zaleski C., Jha S., Batut P., Chaisson M., Gingeras T.R. (2013). STAR: ultrafast universal RNA-seq aligner. Bioinformatics.

[83] Danecek P., Bonfield J.K., Liddle J., Marshall J., Ohan V., Pollard M.O., Whitwham A., Keane T., McCarthy S.A., Davies R.M., Li H. (2021). Twelve years of SAMtools and BCFtools. GigaScience.

[84] Irizarry R.A., Bolstad B.M., Collin F., Cope L.M., Hobbs B., Speed T.P. (2003). Summaries of Affymetrix GeneChip probe level data. Nucleic Acids Res..

[85] Reiner A., Yekutieli D., Benjamini Y. (2003). Identifying differentially expressed genes using false discovery rate controlling procedures. Bioinformatics.

[86] Liberzon A., Birger C., Thorvaldsdóttir H., Ghandi M., Mesirov J.P., Tamayo P. (2015). The Molecular Signatures Database (MSigDB) hallmark gene set collection. Cell Syst..

[87] Gowen B.G., Chim B., Marceau C.D., Greene T.T., Burr P., Gonzalez J.R., Hesser C.R., Dietzen P.A., Russell T., Iannello A. (2015). A forward genetic screen reveals novel independent regulators of ULBP1, an activating ligand for natural killer cells. Elife.

