# Supplementary material for: Ribosomal protein deficiencies linked to Diamond-Blackfan anemia induce distinctive alterations of ATF4 expression
Source: iScience. 2025 Mar 1;28(4):112138. doi: 10.1016/j.isci.2025.112138 (PMC12096137; doi:10.1016/j.isci.2025.112138)
Supplement: Document S1. Figures S1–S5 and Tables S2 and S3 [file mmc1.pdf]

iScience, Volume *28*

Supplemental information

Ribosomal protein deficiencies linked to Diamond-Blackfan anemia induce distinctive alterations of ATF4 expression

L. Francisco Lorenzo-Martín, Javier Robles-Valero, Rosa Ramírez-Cota, Sonia G. Gaspar, Pedro Fuentes, Antonio Gentilella, Xosé R. Bustelo, and Mercedes Dosil

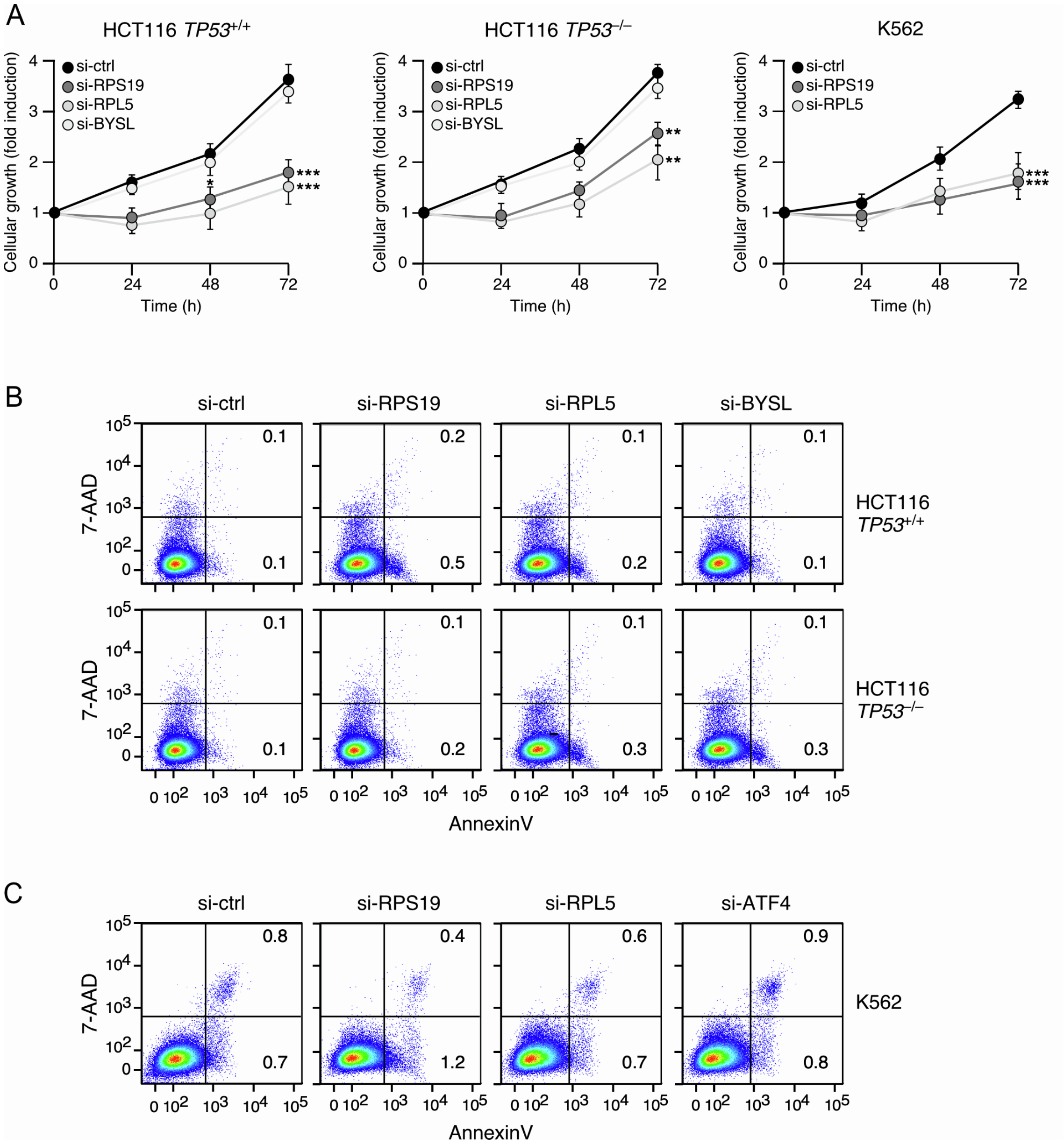

Figure S1. Cell proliferation and viability of HCT116 and K562 cells individually transfected with the si-RNAs employed in this study, Related to Figure 1. (**A**) Effect of indicated si-RNAs in the growth of HCT116 and K562 cells at the indicated time points. Values for each condition and time point are relative to the number of cells at time 0 transfected with the si-ctrl (which was given an arbitrary value of 1). Data represent the mean ± SEM. Statistical values were obtained using the unpaired two-tailed Student's t-test. *P* values are given relative to cells transfected with si-ctrl. **, $P \leq 0.01$; ***, $P \leq 0.001$. n = 3.

(B, C) Flow cytometry detection of apoptotic levels (annexinV-positive cells) in HCT116 (B) and K562 (C) cells at 48 h after transfection with the indicated si-RNAs (n = 3).

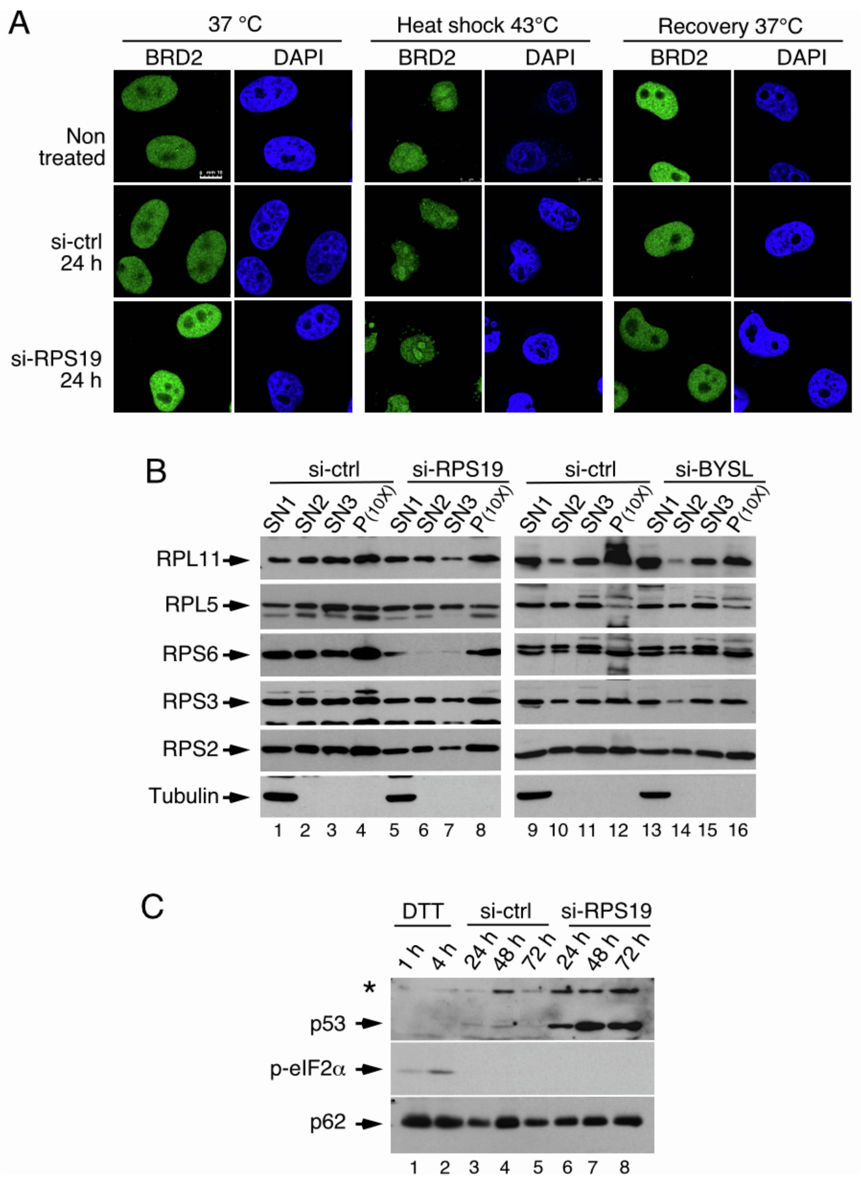

Figure S2. Normal nucleolar sequestration capacity and no signs of proteotoxicity in cells depleted of RPS19, Related to Figure 1. (**A)** Sequestration of the nucleoplasmic protein BRD2 in the nucleolus upon heat stress. HCT116 cells, either untreated or at 24 h after transfection with the indicated si-RNAs, were incubated at 43ºC for 1 h and then allowed to recover at 37ºC for 1 h. Immunostaining of BRD2 was performed in cells non-treated (left panel), just after the heat stress (middle panel), and after the recovery time (right panel). Nuclei were stained with 4',6-diamidino-2-phenylindole) (DAPI). (**B)** PSE solubilization profiles of ribosomal proteins in cells deficient for either RPS19 or bystin.

Western blot analyses showing the contents of the indicated ribosomal proteins and loading control (tubulin) in extract fractions (SN1, SN2, SN3) and insoluble pellet material (P) obtained with the PSE sequential extraction method from HCT116 cells transfected with the indicated si-RNAs and harvested 24 h (samples 1-8) and 48 h (samples 9-16) after transfection. The samples of insoluble material (P) are 10 times concentrated relative to those of the other fractions. **(C)** Analysis of activation of the integrated stress response (ISR) in RPS19-deficient cells. Western blot analyses showing the contents of p53, phosphorylated eIF2-α (p-eIF2α) and p62 in HCT116 cells treated with 5 mM DTT for the indicated times (lanes 1 and 2), and in HCT116 cells transfected with the indicated si-RNAs and harvested at the indicated times after transfection (lanes 3-8). The asterisk in the first panel indicates an unspecific band that is detected with the p53 antibody.

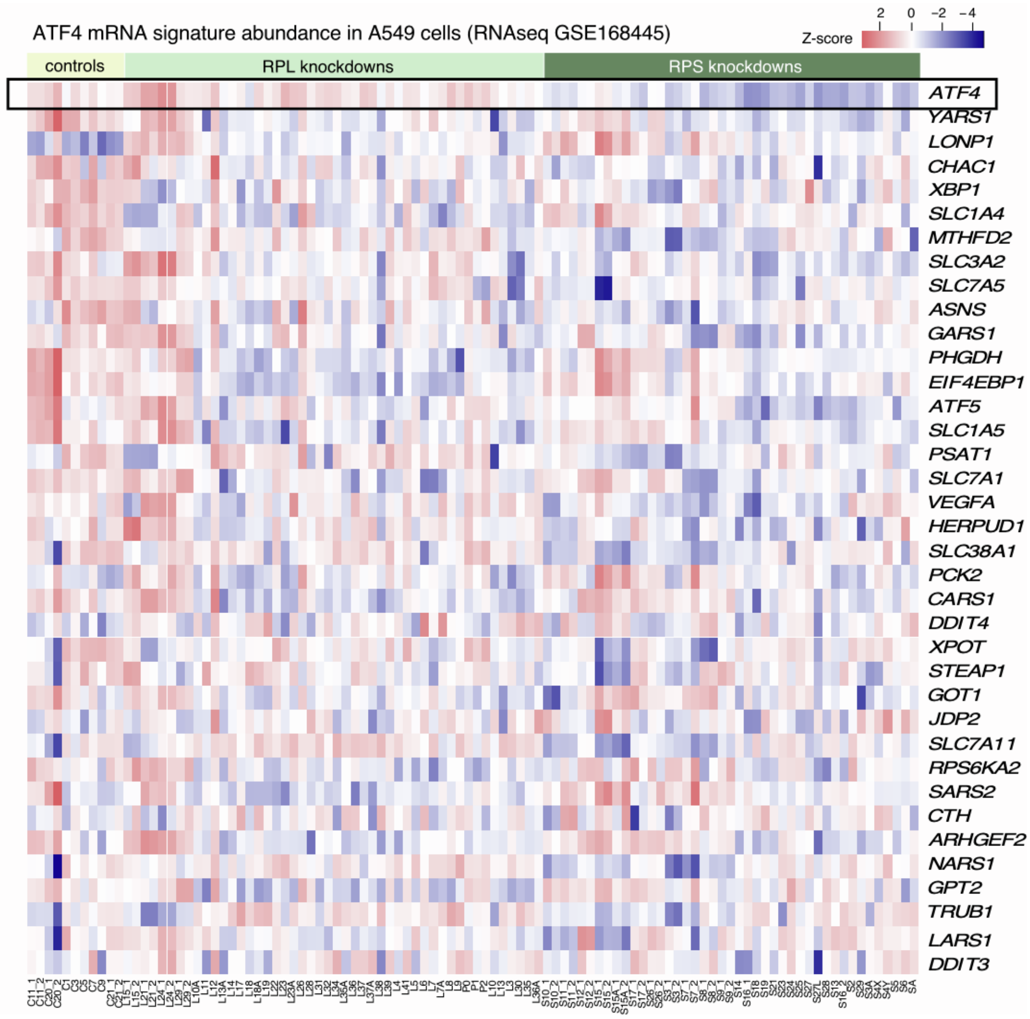

Figure S3. Relative abundance of ATF4 and AmTOR signature mRNAs in RPS and RPL knockdown cells, Related to Figure 5. Heatmap of ATF4 and AmTOR signature transcript contents in A549 cells after the individual siRNA-mediated knockdown of 75 ribosomal proteins (31 RPSs and 44 RPLs). The values of ATF4 and AmTOR signature mRNA levels were retrieved from the GSE168445 RNA-seq dataset. Columns represent the individual knockdowns of ribosomal proteins (names indicated at the bottom). The gene expression analyses totally segregate the RPS and RPL knockdowns. The most distinctive feature that separates the two groups is the abundance of ATF4 mRNA, which is reduced in the RPS knockdowns group as compared to the control and RPL knockdowns group. The row corresponding to ATF4 mRNA is indicated. For simplicity,

the ATF4 row is the only one shown in main Figure 4A. Relative differences in mRNA abundances are represented according to the Z-score color scale shown at the top.

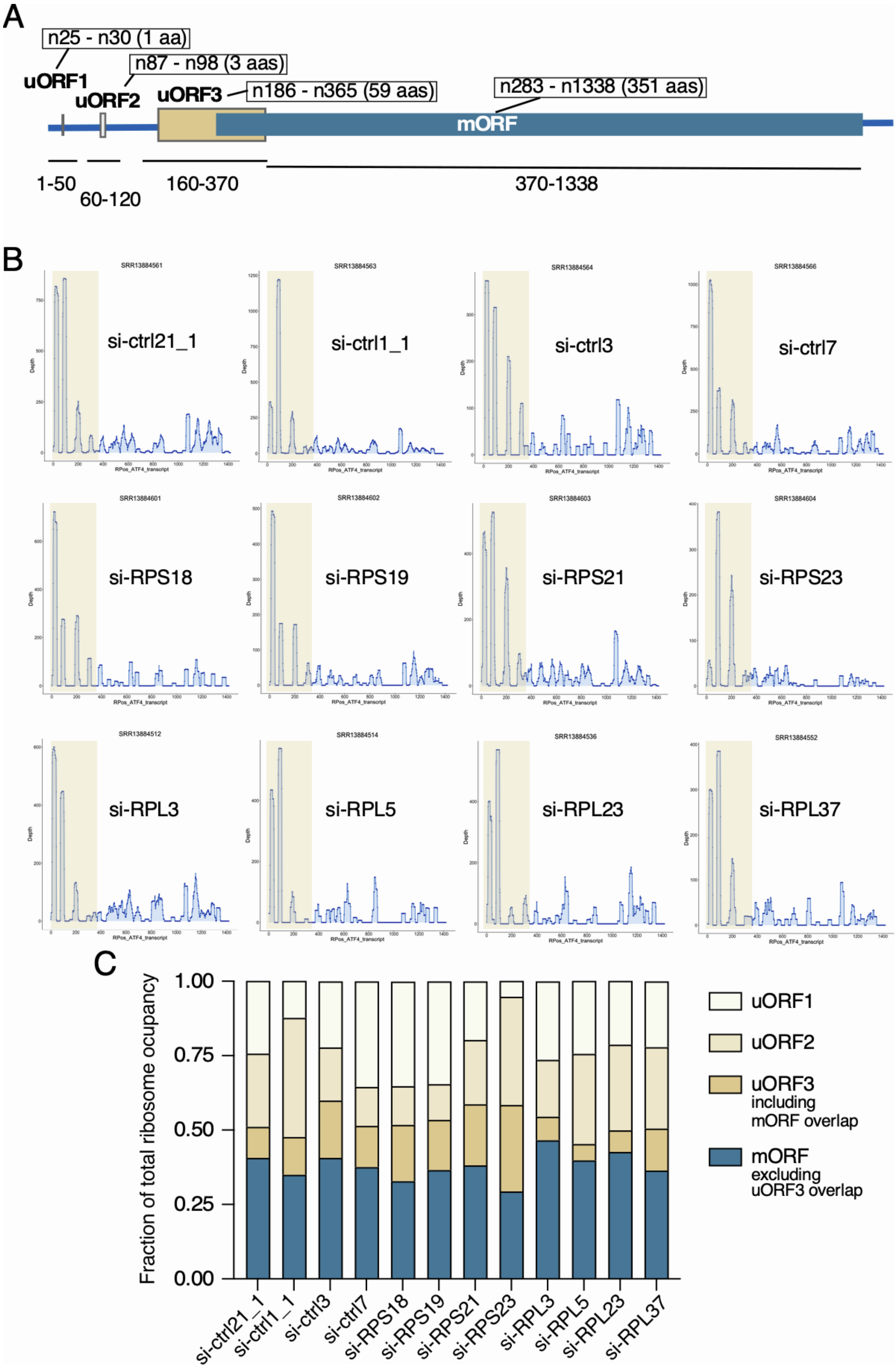

A
n25 - n30 (1 aa)
n87 - n98 (3 aas)
uORF1
uORF2
uORF3
n186 - n365 (59 aas)
n283 - n1338 (351 aas)
mORF
1-50
60-120
160-370
370-1338
B
si-ctrl21_1
si-ctrl1_1
si-ctrl3
si-ctrl7
si-RPS18
si-RPS19
si-RPS21
si-RPS23
si-RPL3
si-RPL5
si-RPL23
si-RPL37
C
Fraction of total ribosome ocupancy
1.00
0.75
0.50
0.25
0.00
uORF1
uORF2
uORF3 including mORF overlap
mORF excluding uORF3 overlap
si-ctrl21_1
si-ctrl1_1
si-ctrl3
si-ctrl7
si-RPS18
si-RPS19
si-RPS21
si-RPS23
si-RPL3
si-RPL5
si-RPL23
si-RPL37

Figure S4. Ribosome occupancy distribution on the ATF4 transcript in RPS and RPL knockdown cells, Related to Figure 5. **(A)** Diagram of the ATF4 transcript showing the positions and nucleotides encompassed by the three uORFs and the mORF. The lines and numbers at the bottom of the graph indicate the extensions and coordinates of the four regions used for the fractional quantitations of ribosome occupancy shown in main Figures 4F and 4G, and in this figure in panel **C**. The first region contains uORF1, the second contains uORF2, the third contains uORF3 (including the overlap with the mORF), and fourth contains mORF excluding the overlap with uORF3. **(B)** Ribosome occupancies on the ATF4 transcript in A549 cells transfected with the indicated si-RNAs. RPF reads were retrieved from the GSE168445 Ribo-seq dataset and assigned to their corresponding positions on the ATF4 mRNA. The shaded region in each graph corresponds to the 5'-UTR plus the 5'-region of the mORF that overlaps with uORF3. **(C)** Relative ribosome occupancies at four consecutive regions of the ATF4 transcript (indicated in **A**) in A549 cells transfected with the indicated si-RNAs.

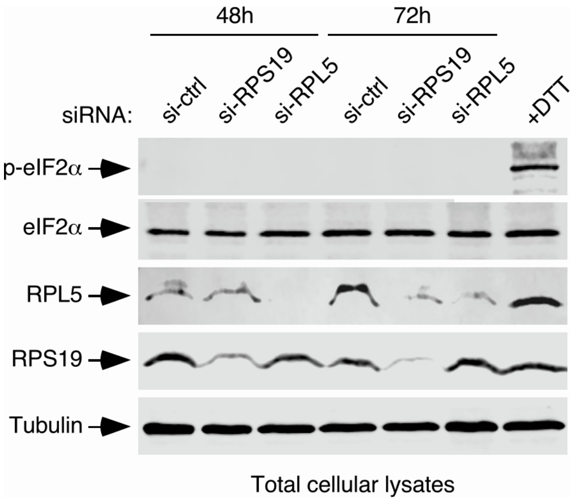

Figure S5. Analysis of activation of the integrated stress response (ISR) in RPS19- or RPL5-deficient K562 cells, Related to Figure 7. Western blot analysis showing the contents phosphorylated eIF2-α (p-eIF2α), total eIF2-α, RPL5, RPS19 and tubulin in total cellular lysates of K562 cells transfected with the indicated si-RNAs and harvested at the indicated times after transfection. Cells treated with 5 mM DTT for 2 h were included as a positive control.

Table S2. siRNAs used in this study.

siRNA	Gene symbol	Gene name	Gene ID	siRNA ID	Sense siRNA sequence
si-BYSL	BYSL	Bystin-like	705	s2134	5’-CCAGGAUUUUUGCCUCUAAtt-3’
si-RPL5	RPL5	Ribosomal protein L5	6125	Custom	5’-CUACCACUGGCAAUAAAGUtt-3’
si-RPS19	RPS19	Ribosomal protein S19	6223	Custom	5’-UGGCGGCCGCAAACUGAUUtt-3’

Table S3. List of oligonucleotides used in this study. Fw, forward; Rv, reverse.

Oligonucleotide	Sequence	Use
5’ITS1	5’-CCTCGCCCTCCGGGCTCCGTTAATGATC-3’	Northern blot
ATF4 Fw	5’-TTCTCCAGCGACAAGGCTAAGG-3’	RT-qPCR
ATF4 Rv	5’-CTCCAACATCCAATCTGTCCCG-3’	RT-qPCR
CDKN1A Fw	5’-GCCGAAGTCAGTTCCTTGTGGA-3’	RT-qPCR
CDKN1A Rv	5’-GTCACCCTCCAGTGGTGTCT-3’	RT-qPCR
CHAC1 Fw	5’-GTGGTGACGCTCCTTGAAGATC-3’	RT-qPCR
CHAC1 Rv	5’-GAAGGTGACCTCCTTGGTATCG-3’	RT-qPCR
HERPUD1 Fw	5’-CCAATGTCTCAGGGACTTGCTTC-3’	RT-qPCR
HERPUD1 Rv	5’-CGATTAGAACCAGCAGGCTCCT-3’	RT-qPCR
